# Monodisperse Condensed Tannin and Derived Carbon Nanospheres Featuring Well‐Defined Surface‐Structured Metal Species

**DOI:** 10.1002/smll.75461

**Published:** 2026-08-26

**Authors:** Weitong Wang, Jiaqi Wang, Bin Zhao, Tianyang Wen, Daniel Martín‐Yerga, Monika Österberg, Erlantz Lizundia, Bruno D. Mattos

**Affiliations:** ^1^ Department of Bioproducts and Biosystems School of Chemical Engineering Aalto University Espoo Finland; ^2^ Department of Chemistry and Materials Science School of Chemical Engineering Aalto University Espoo Finland; ^3^ Department of Applied Physics School of Science Aalto University Espoo Finland; ^4^ Department of Chemical and Metallurgical Engineering School of Chemical Engineering Aalto University Espoo Finland; ^5^ Life Cycle Thinking Group Department of Graphic Design and Engineering Projects Faculty of Engineering in Bilbao University of the Basque Country (EHU) Bilbao Spain

**Keywords:** electrocatalysis, metal‐phenolic networks, nanoparticles, polyphenols, proanthocyanidins

## Abstract

Direct synthesis of monodispersed nanospheres from heterogeneous biopolymers remains a current challenge within materials science. Here, we introduce the assembly of uniform and size‐tunable condensed tannin (proanthocyanidin) nanospheres (TNSp) via a hydrogen bonding‐driven hydrophobic assembly that takes place in aqueous medium and ambient conditions. Uniform particles are formed by a gradual increase in tannin‐amine concentrations in the reaction system, which regulates nucleation and growth via supramolecular interactions. The phenolic‐amine rich surface of the nanospheres allows a precise structuring of metal species (*e.g*., Pt, Co, Ni), thus ensuring high dispersion, uniform particle size, and suppressed sintering of the metal species (1–2 nm) during pyrolysis. Derived carbon particles with surface‐anchored Pt nanoparticles (Pt‐TNSpC) are then demonstrated as high performance catalysts in the electro‐hydrogenation of guaiacol, holding performance surpassing that of Pt/C catalyst. Nevertheless, life cycle assessment revealed a significantly lower carbon footprint against benchmark catalysts (1770 kg CO_2_‐eq.·kg^−1^ vs. 11510–82660 kg CO_2_‐eq.·kg^−1^), alongside enhanced material circularity. This work establishes a sustainable strategy for converting renewable biobased feedstocks into precision nanostructured particles for high performance catalysis. We envision these metal‐functionalized tannin‐derived particles to find use in various fields in which metallic functionalities play a role.

## Graphical abstract

Uniform, size‐tunable polyphenol nanospheres are assembled from condensed tannins in water at ambient conditions via controlled supramolecular interactions between tannin and diamines. Their phenolic–amine surfaces template highly dispersed metal species that resist sintering during pyrolysis. Resulting metal‐functionalized carbon particles outperform benchmark catalysts in guaiacol electrohydrogenation, while displaying substantially lower environmental impact and enhanced circularity.

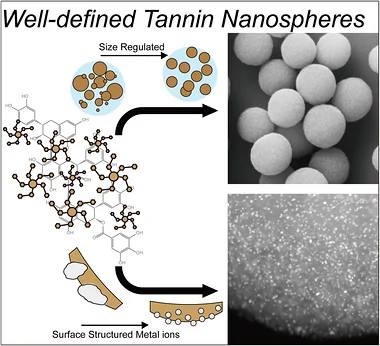

## 1 Introduction

Polymeric colloidal nanospheres are sought for applications in photonics, biomedicine, energy, and electrocatalysis, with their performance strongly depending on size, dispersity, and surface functionality [1, 2]. These nanospheres are commonly synthesized from poly(styrene), poly(methyl methacrylate), and phenolic resins [3, 4, 5] because these precursors are chemically well‐defined and pure, thus enabling precise control over reaction kinectis and consequently particle growth. Uniformity – dimensional and morphological – is a key feature across several nanotechnologies, as it governs surface interactions and associated assemblies. For instance, highly uniform nanospheres can self‐assemble into three‐dimensional ordered structures that exhibit unique optical properties and that offer control over mass diffusion in electrocatalysis [6, 7]. Despite their excellent monodispersity, poly(styrene) and poly(methyl methacrylate) nanospheres suffer from poor thermal stability, limiting their conversion into functional carbon materials [8]. Nanospheres derived from phenolic resins offer superior thermal and mechanical stability; however, their synthesis heavily relies on energy‐intensive polymerization processes and petroleum‐based reagents that raise significant environmental concerns [9, 10]. Performance and low environmental impact have been almost entirely mutually exclusive.

Plant‐derived polyphenols are abundant [11] and their corresponding materials have been increasingly explored in fields traditionally dominated by fossil‐derived polymers [12, 13]. Lignin nanospheres (LNPs) have been extensively studied over the past decades. However, the most established fabrication methods, including emulsion templating and solvent‐shifting strategies, are multistep and require large quantities of organic solvents [14, 15]. Lignin tends to yield soft particles that easily lose their structural integrity under conventional pyrolysis, limiting their use in applications where well‐defined carbon micro‐/nanostructures are essential (e.g., catalysis) [16]. Condensed tannins, particularly proanthocyanidins abundant in bark tissues and shrubs, are smaller polyphenols when compared to lignin, more reactive, and display high binding capacity that enables their functionalization with metal species. Such features make them promising candidates for the manufacturing of particulate functional materials. Hydrothermal processing has been employed to make particles from condensed tannins, but this process goes from dissolved molecules directly into carbon particles thus leading to inert surfaces that cannot be easily modified for functions [17]. Other strategies combine condensed tannins with synthetic phenolic compounds to form hybrid nanospheres, which compromises the biobased content of the particle while still providing limited control over particle size and uniformity [18, 19, 20]. Therefore, a simple and straightforward method for producing well‐defined functionalizable nanospheres from condensed tannin (e.g. proanthocyanidins) remains unreported.

Most biocolloid assemblies are driven by noncovalent interactions, such as hydrophobic effects, π–π stacking, hydrogen‐bonding, and van der Waals forces, which collectively stabilize supramolecular aggregates [21, 22]. However, the intrinsically complex and polydentate structures of polyphenols often lead to uncontrolled crosslinking and aggregation during particle formation. In amine‐containing systems, polyphenols readily undergo deprotonation, while the released protons simultaneously enhance amine protonation. This mutual activation markedly accelerates hydrogen‐bonding‐driven strategies, frequently leading to rapid and poorly controlled nucleation and particle growth [23]. As a result, the obtained nanospheres typically exhibit broad particle‐size distributions and significant polydispersity. Such structural heterogeneity also poses a major challenge for the precise spatial organization of metal nanoparticles on the particle surfaces. Spatially localizing metal nanoparticles is perhaps the most difficult challenge within the designing of metal‐anchored materials for heterogeneous catalysts. Well‐defined support structures can confine metal nanoparticles, enabling homogeneous dispersion, suppressing agglomeration, and preventing sintering during high‐temperature treatments [24, 25]. While polyphenols have strong metal‐chelating capabilities, the absence of a well‐defined nanostructure hinders precise surface structuring of metal nanoparticles.

Here, we report a facile aqueous strategy for the direct synthesis of uniform and tailorable tannin nanospheres (TNSp) from condensed tannins. Our process is based on controlling supramolecular interactions among condensed tannins molecules and amines employed to trigger the particle formation (Figure 1a). Uniform, and size‐tunable particles, are formed by making a gradual enrichment of tannin–amine species in reaction system for an effective regulation of nucleation and particle growth. The phenolic–amine‐rich surface microenvironment further enables uniform anchoring of Pt species (and others) through strong metal–polyphenol interactions, producing highly dispersed active sites after carbonization. Unlike conventional post‐impregnation of metal species in carbon supports, this system allows precursor‐level integration of metal species and direct carbonization of such metal‐polyphenol particles into high performance catalysts. We demonstrate the effects of well dispersed Pt species on uniform tannin‐derived carbon nanospheres in the electro‐hydrogenation of guaicol. Moreover, owing to the renewable feedstocks and mild synthesis conditions, the resulting catalyst exhibits a dramatically reduced carbon footprint (∼1.7 tons CO_2_‐eq·kg^−^^1^), ∼40 times lower than benchmark noble‐metal catalysts.

**FIGURE 1:**
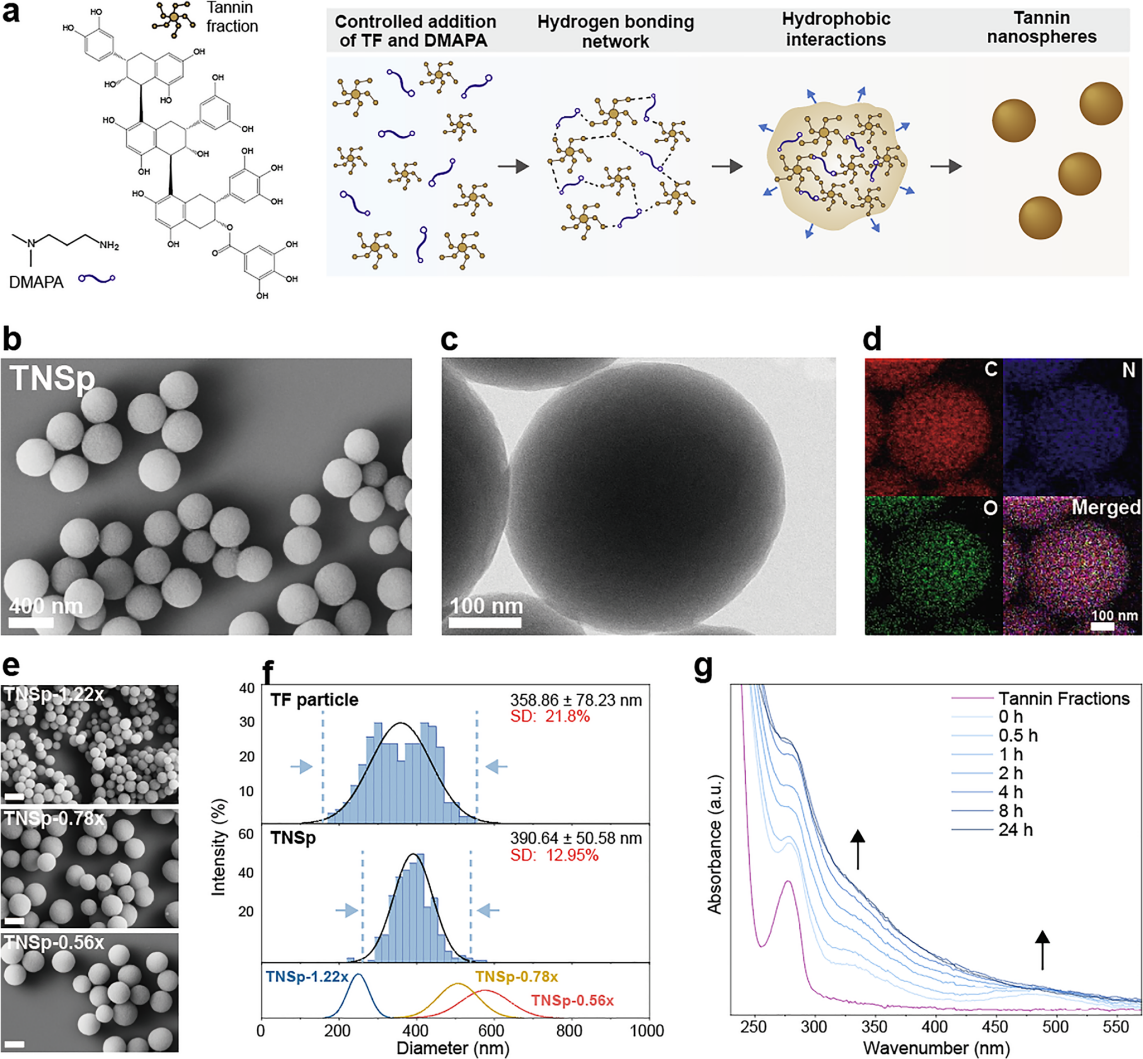
Preparation and morphology of the nanospheres. (a) Schematic of the hydrogen‐bonding induced self assembly of tannin nanospheres (TNSp). (b) Scanning (SEM) and (c) transmission electron microscope (TEM) images of the TNSp, and (d) Energy Dispersive x‐ray Spectroscopy (EDS) elemental mapping acquired in SEM. (e) SEM micrographs of particles different diameters obtained by adjusting the solution volume to 0.56, 0.78, and 1.22 times than the typical system (0.56x, 0.78x, and 1.22x, scale bar = 400 nm). (f) Particle size distribution and standard deviation of nanospheres prepared in different volumes (SD: standard deviation). (g) Time‐resolved TNSp formation tracked by UV–vis spectroscopy.

## 2 Results and Discussion

### 2.1 Fabrication of Proanthocyanidin Particles via Controlled Supramolecular Interactions

Polyphenol‐based micro/nanoparticles typically require a dissolution–regeneration process, with particle properties dictated by molecular size and solubility of the precursors [26, 27]. Based on our prior work [28], the fractionated condensed tannin (TF), i.e. proanthocyanidins, display improved homogeneity and solubility (Figure S1). Uniform tannin nanospheres (TNSp) were assembled directly in aqueous, at room temperature, using dimethylaminopropylamine (DMAPA) as a hydrogen‐bonding trigger with fractionated tannin (Figure 1a–c), while unfractionated condensed tannin led to irregularly shaped particles (Condensed tannin particles, CTPs, Figure S2). Another key step for making well‐defined nanospheres was to control the addition rate of the DMAPA and TF solutions into the reaction system. The aggregation initiated by intermolecular hydrogen bonds was fast and caused an uneven growth of TF particle with a broad size distribution (Figure S3). Preparation of uniform spheres from the rapid reaction kinetics between phenolics and diamines has been reported, but this method works only for synthetic phenolic resins [8]. Therefore, we controlled the addition rate (at 4 mL/min) of TF and DMAPA solutions to achieve a slow increase in the concentration to control hydrogen bonding kinetics between them (Figure S4), thus ultimately resulting in highly uniform nanospheres.

The TNSp produced with slow precursor addition are well‐shaped monodisperse spheres, with a remarkable improvement in dispersity (from 20% to 10% deviation) when compared to particles produced with immediate mixture of the two precursors (Figure 1e,f and Figure S5), as well as particle yields of *ca*. 50%. TNSp of various well‐defined sizes and deviation *ca*. 10% could be obtained by adjusting the reaction volume (0.56, 0.78, and 1.22 times relative to the typical system). In that case, the particle size correlated negatively with the reaction volume. In these experiements, the absolute amounts of tannin and amine were fixed while varying only the solvent (water) volume. Therefore, increasing the reaction volume corresponded to a decrease in reactant concentration. According to the classical LaMer nucleation–growth model, the final particle size is determined by the competitive relationship between the nucleation and growth stages. At lower reactant concentrations (*i.e*., in the larger‐volume system), the number of initial nuclei formed is relatively small; however, the monomer supply available for subsequent growth of these few nuclei is also limited, resulting in suppressed growth and ultimately smaller nanoparticles. In contrast, at higher reactant concentrations (*i.e*., in the smaller‐volume system), a large number of nuclei are generated during the nucleation burst, and the abundant monomer supply readily supports the subsequent growth of these nuclei, leading to larger particles.

To better understand the mechanism of particle formation, we first time‐resolved the particle assembly process via UV–vis spectroscopy. UV–vis spectroscopy (Figure 1g) revealed that during the assembly process of TNSp, the characteristic peak of tannin at 278 nm gradually decreased during assembly, while new peaks at 326 and 482 nm appeared and increased in intensity as the reaction progressed. Both peaks emerge from charge transfer effects induced by hydrogen bonding and associated (de)protonation events (Figure S6) [29, 30]. These spectral changes were more pronounced within the first hours of reaction and diminished after 4 h, indicating the completion of the assembly and its gradual nature (Figure S7). Energy‐dispersive x‐ray spectroscopy (EDS) mapping and x‐ray photoelectron spectroscopy (XPS) analysis confirmed that TNSp consisted of C, O, and N elements (Figure 1d and Figure S8). Fourier‐transform infrared spectroscopy (FTIR) spectra revealed a broadened O─H stretching band at *ca. *3300 cm^−1^, and no significant changes from 1700 to 1000 cm^−1^ region, ruling out the formation of amide bonds during the particle formation (Figure S9). Moreover, there was not major differences in the thermogravimetric profiles of the TNSp when compared to the tannin fractions (Figure S10). All these results indicate a non‐covalent assembly pathway.

To further elucidate the particle formation mechanism, we performed ^1^H NMR of the resulting TNSp and its primary components, as well as MD simulations of model systems. Complementary experiments were performed using analytical grade epigallocatechin gallate (EGCG) as a reference molecule, as it is a structurally well‐defined polyphenol that undergoes similar supramolecular interactions compared to our condensed tannin fractions (Figures S11–S14). NMR analysis revealed the disappearance of the characteristic phenolic hydroxyl signal at 8—10 ppm and the emergence of a broadened signal at 4.58 ppm, indicating deprotonation of peripheral phenolic hydroxyl groups and the formation of a dense hydrogen‐bonding network [O─H···N] between tannin and DMAPA (Figure 2a) [31]. The observed broadening signal originates from rapid proton exchange within this dynamic [O─H···N] involving multiple intermediate states, while the absence of signal in the corresponding region of the 2D HSQC spectrum confirms the non‐covalent interactions (Figure S14). Variable‐temperature ^1^H NMR spectroscopy showed the broad phenolic proton signal shifting high‐field (from δ = 4.58 ppm to δ = 4.35 ppm, Δδ = ‐0.23 ppm) with increasing temperature to 70°C, which further supports a dynamic [O─H···N] with rapid proton exchange between tannin and DMAPA [32]. Besides, DMAPA does not undergo covalent changes upon assembly, as evidenced by only minor chemical shifts of its characteristic signals at δ = 1.44, 2.19, 2.52 ppm by minor Δδ = 0.03, 0.02, 0.03 ppm after assembly, and slightly low‐field shifts of Δδ = 0.05, 0.06, 0.08 ppm with increasing temperature. This corroborates with the signals of protonated amines (‐NH_3_^+^) obtained by XPS of TNSp (Figure S8). These NMR behaviors were even more distinctly manifested in an EGCG‐DMAPA system, where the well‐defined molecular structure provided clearer insights into the dynamic assembly process (Figure S14).

**FIGURE 2:**
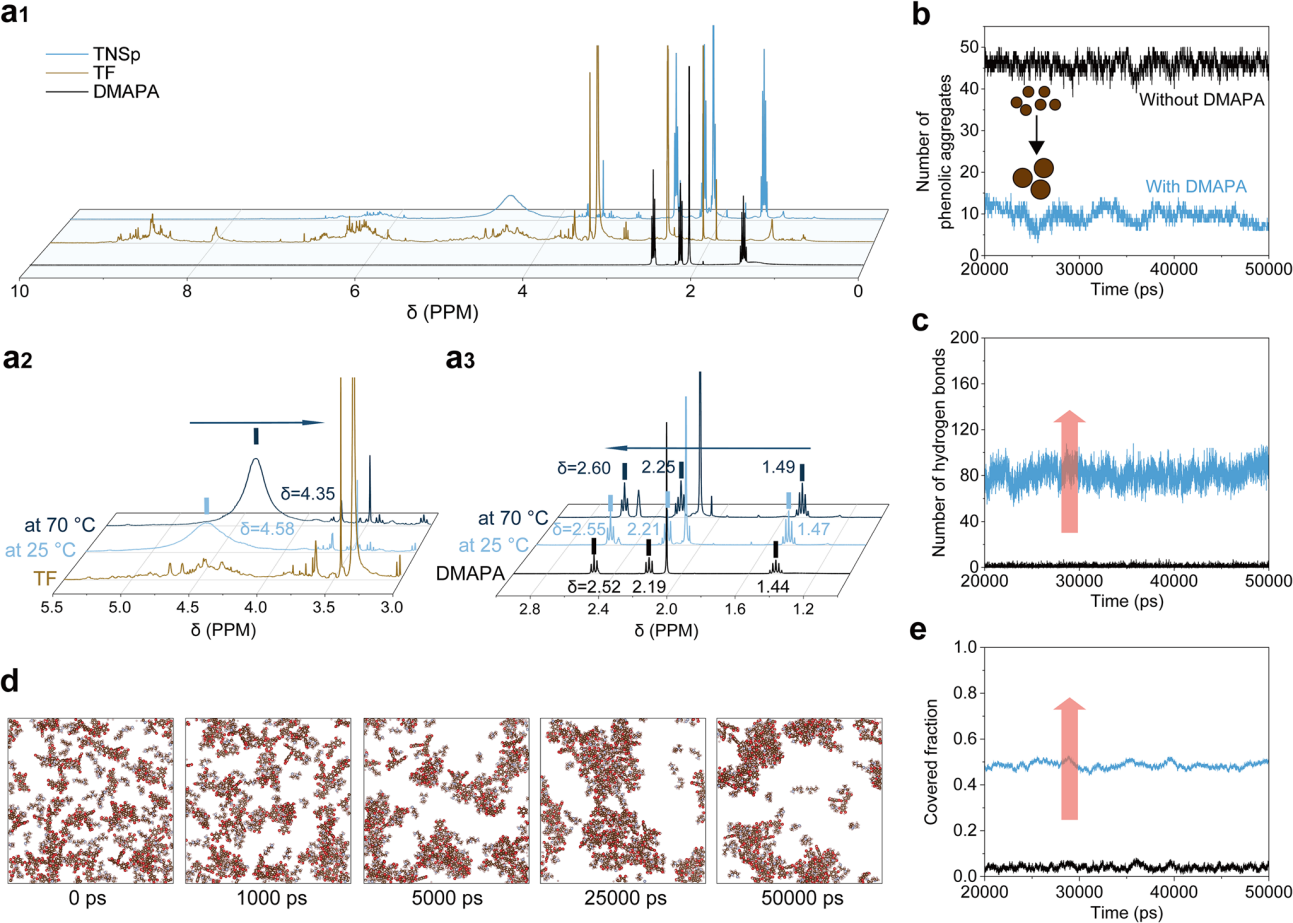
Particle formation mechanism. (a1) ^1^H NMR spectra of different components in reaction complexes and TNSp, as well as a detailed spectra of TNSp perform under different temperature compared with (a2) tannin fraction and (a3) DMAPA at 25°C. (b) Number of aggregated phenolic as a function of time. (c) Number of hydrogen bonds in different simulated tannin particle formation systems – with and without DMAPA. (d) Evolution state of the model phenolic‐DMAPA complex, primarily driven by intra‐ and intermolecular hydrogen bonding. (e) Covered fraction (the fraction of molecules not accessible to the solvent) of phenolic molecules.

Through MD simulations, we further analyzed the hydrogen bonding interactions that are dominant during the particle assembly process (Figure 2b). The structural units of the phenolic‐diamine aggregates are stabilized by dynamic [O─H···N] interactions. Here, EGCG was selected as a model compound to represent TF (Figure S15). We compared the number of hydrogen bonds in EGCG‐DMAPA and EGCG‐solely systems under identical solvent conditions. The results demonstrate a significant increase in hydrogen bond formation in the presence of DMAPA (Figure 2c and Figures S16 and S17), whereas in systems containing only EGCG, intermolecular hydrogen bonding and other non‐covalent interactions are insufficient to induce aggregation. Importantly, replacing DMAPA with ethylenediamine (EDA), which contains two primary amine groups and a shorter alkyl chain, did not substantially alter the number of hydrogen bonds (Figure S18). Both simulation and experimental results confirmed that EDA could not successfully self assemble with model phenolics (Supplementary Figures S11 and S18). This suggests that while hydrogen bonding provides essential directional attraction, the hydrophobic contribution of the alkyl chain in DMAPA plays a critical role in enabling the polymers to phase‐separate from water after the initial hydrogen‐bonding aggregates forms and creates seed particles that possess segments inacessible to solvent, which increases in the presence of the hydrophobic interactions provided byf DMAPA (Figure 2e).

### 2.2 Phenol‐Driven Networking of Metal Species on the Particle Surface

Our supramolecular TNSp nanospheres possess abundant surface‐exposed catechol and galloyl groups, which are known for their strong metal‐networking capabilities [33]. These phenolic ligands can bind various transition metals as well as reduce noble metal ions, thereby anchoring metal species and stabilizing them at the surface of the hosting TNSp (Figures S19–S23). Furthermore, the amine groups introduced during preparation may enhance metal adsorption and dispersion through synergistic interactions with phenolic motifs [19], although amines are primarily consumed during the nanoparticle formation step. The primary advantage of our particles is that it establishes a wide range of possibilities by using a single starting particle host. Herein we decorated TNSp with Pt species due to their catalytic properties, but we also demonstrate the suitability of the method for Co and Ni, and we envision that any other positively charged metal species could be introduce in the same manner. Such Pt, Co and Ni species interact differently with the surface‐exposed phenolic motifs on TNSp (Figure 3a). UV–vis spectra revealed a pronounced ligand‐to‐metal charge transfer (LMCT) band at 400–500 nm for Co and Ni systems, indicative of the formation of metal‐phenolic periodic complexes (Figure 3b) [24]. On the other hand, Pt species undergo direct reduction to their metallic state, following a redox pathway, as evidenced by the absence of a clear LMCT broad band (Figure 3c) [34]. In the case of anchored Pt ions, the PtCl_4_ precursos forms anionic complexes that are captured on TNSp via coordination to abundant phenolic hydroxyls. These phenolic groups both anchor Pt (IV) and reduce it in situ to Pt (0) (oxidizing to quinones), enabling a one‐step capture–reduction process. Unlike deposition–precipitation, no external precipitants or H_2_ reduction are needed, yielding highly dispersed Pt nanoparticles after carbonization.

**FIGURE 3:**
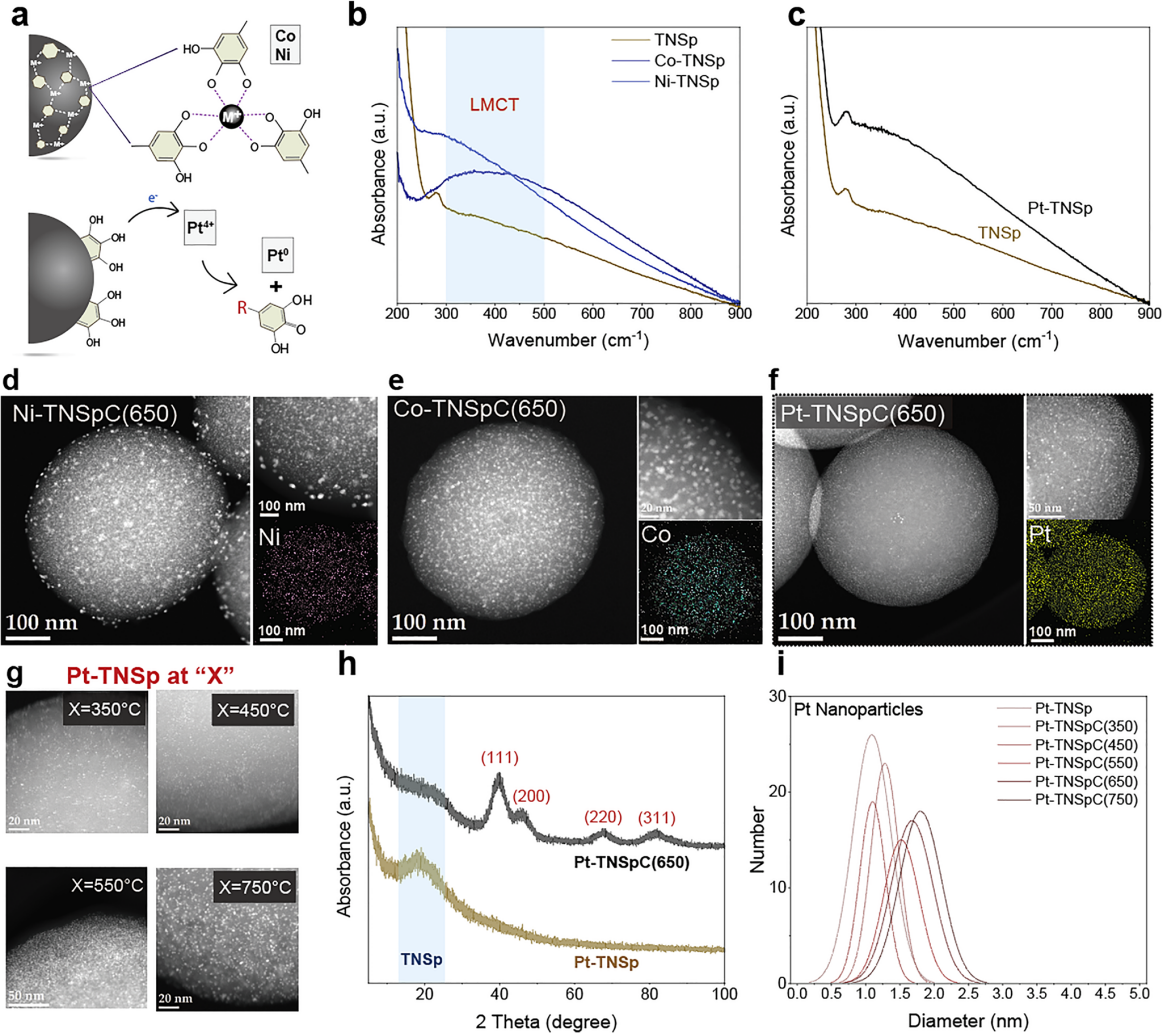
Metal‐functionalization of tannin particles, and carbonization. (a) Schematics representing the two main mechanisms of metal binding by surface exposed phenol motifs. UV–vis spectroscopy of (b) TNSp, Co‐TNSp, and Ni‐TNSp exhibiting clear ligand‐to‐metal charge transfer (LMCT) effect and (c) TNSp and Pt‐TNSp displaying no LMCT features. TEM images followed by EDX mapping of (d) Ni‐TNSpC(650), (e) Co‐TNSpC(650), and (f) Pt‐TNSpC(650) which is further used in all catalytic experiments. (g) Effect on the pyrolysis temperature on the morphology of the Pt‐TNSpC catalystis (scale bar = 25 nm). (h) XRD patterns of Pt‐TNSpC(650) and Pt‐TNSp. (i) Size distribution of Pt nanoparticles on the surface of TNSpC pyrolized at various temperatures.

Then we proceeded with pyrolysis of the metal‐coated TNSp carbon hosting particles. Whereas hydrogen bonds are key to initiate the assembly and to establish an ordered precursor architecture, they are then dissociated upon heating given place to the ordered (and dense) formation of other interactions within the precursor—such as π–π stacking between aromatic rings and the progressively formed crosslinked networks— gradually “locking in” the spherical morphology during carbonization. Notably, the crosslinked nature of our TNSp enabled us to skip the extensive thermal stabilization step (e.g. 250°C for 2 h at 0.3°C/min, totalling > 10 h) that is required to maintain the morphology of other natural polyphenol particles (e.g. lignin) during carbonization [16, 35]. Darkfield transmission electron microscopy (TEM), along with EDS elemental analysis, revealed the presence of uniformly distributed fine metal nanoparticles on the surface of the hosting TNSpC (Figure 3d–f). All three metal species tested were stable and uniform, even if interacting differently with the hosting TNSp. This highlights the versatile metal‐binding ability of tannin molecules, arising from their polydentate nature, which presents multiple phenolic binding sites.

Moreover, we tested the stability of Pt species onto TNSp under different pyrolysis temperatures. Pt species greatly resisted coalescence, maintaining a well‐dispersed state across all tested temperatures. Sizes of surface Pt nanoparticles were quantified in high‐resolution TEM images (Figure 3f,g, Figure S24), revealing that the average particle size increased by only ∼0.7 nm from 350°C to 750°C (Figure 3i). TNSp showed carbon yields from 69.5% at 350°C to 48.7% at 750°C, while maintaining monodisperse spherical morphology. The nanospheres reduced in size by only 19.7% at 350°C and 450°C, with subsequent shrinkage of *ca*. 20%, 30%, and 43% at 550°C, 650°C, and 750°C (Figure S25). Overall, the carbonization did not compromise the structural integrity of the nanospheres, underscoring the robustness of TNSp hosting architecture. The significantly higher dispersion of Pt compared to Co and Ni can be attributed to the fundamental difference between “free” nucleation–growth (for Pt) and “constrained” coordination anchoring (for Co/Ni). The former allows Pt to form small, well‐separated particles through surface reduction and migration, whereas the latter results in stronger retention of Co/Ni within the framework, limiting their ability to migrate and coalesce into uniformly dispersed small particles.

XPS further provided definitive evidence of the oxidation‐reduction states of Pt species onto tannin catalysts, confirming the presence of Pt (0) (Figure S23). Moreover, XPS indicates the role of the phenolic motifs on the reduction process, by comparing the percentual content of Pt (0) in the impregnation method (ca. 58%) with our system (ca. 73%). The high‐resolution Pt 4f spectrum exhibited peaks at binding energies of approximately 71.0, 72.8, and 74.1 eV, corresponding to Pt (0), Pt (II), and Pt (IV), respectively. The dominant contribution of Pt (0) (73.02%), with minor contributions from Pt (II) and Pt (IV) species, conclusively established the superior reducing capacity of the phenolic matrix. Finally, x‐ray diffraction (XRD) spectra confirmed the growth of small Pt nanoparticles on the surface of TNSp after the carbonization, revealed by characteristic peaks corresponding to the (1 1 1), (2 0 0), (2 2 0), and (3 1 1) crystal planes (Figure 3h and Figure S26). The complete absence of diffraction peaks characteristic to Pt in the Pt‐TNSp material unambiguously rules out the presence of pre‐existing metallic clusters prior carbonization. The initial distribution of metal species on TNSp is preserved under pyrolysis, thus ensuring a high accessibility of catalytic sites for a superior activity.

### 2.3 Tannin Catalyst for the Electrocatalytic Hydrogenation (ECH) of Lignin‐Derived Compounds

The uniform and robust condensed tannin nanospheres provide a versatile platform for multiple particle‐based catalytic applications. Here we demonstrate the use of tannin catalyst Pt‐TNSpC(650)‐Cat for the electrocatalytic hydrogenation (ECH) of guaiacol, a key lignin‐derived platform chemical as well as a feedstock for the sustainable production of fuels and fine chemicals [36, 37]. To identify optimal reaction conditions, we first performed a two‐step screening: (*i*) evaluation of catalysts prepared at different pyrolysis temperatures, and subsequently (*ii*) study of the working potential for the best‐performing material (Figures S27 and S28). Analysis revealed that Pt‐TNSpC(650)‐Cat and a working potential of −67 mV vs. reversible hydrogen electrode (RHE) provided the optimal conditions, which were therefore selected for comparison with a commercial Pt/C. To highlight the role of Pt dispersion across the catalyst, we prepared a reference material using a conventional metal impregnation method (refered to TNSpC/Pt‐IM, Figure S29).

Under these optimized conditions, Pt‐TNSpC(650)‐Cat outperformed TNSpC/Pt‐IM and Pt/C in guaiacol conversion, product yield and faradaic efficiency (Figure 4a), despite containing 20% lower Pt content (*ca*. 4 wt.%) than Pt/C (5 wt.%) (Figure S25). Our catalyst achieved efficient hydrogenation of the aromatic ring while selectively cleaving the C─O(CH_3_) bond and retaining the C─O(H) bond. Notably, Pt‐TNSpC(650)‐Cat achieved a cyclohexanol selectivity of 74.8%, more than double that of Pt/C (29.6%) (Figure 4b). Pt‐TNSpC(650) also delivered higher (*ca*. 125%) current densities at same applied potential (Figure 4c), reflecting its superior pre‐site activity, or a lower potential at the same current density (Figure 4d). This enhancement does not necessarily reflect intrinsically superior per‐site activity; rather, it arises from a larger population of accessible active sites, as Pt‐TNSpC(650)‐Cat exhibited a higher electrochemically active surface area (ECSA) (Figure 4f and Figure S30), despite its lower Pt loading. This indicates that the Pt is more uniformly dispersed and efficiently utilized across the nanosphere surface, enabling a higher fraction of Pt atoms to participate in the reaction. In short, the surface structuring across the tannin‐derived nanospheres provided enhanced metal atom utilization compared to state‐of‐the‐art commercial Pt/C catalysts or catalysts prepared by impregnation methods. The performance of the present catalyst also compares with representative literature‐reported catalysts for guaiacol electrocatalytic hydrogenation (Table S1). Overall, Pt‐TNSpC(650)‐Cat provides a favorable balance between reaction rate, cyclohexanol selectivity, and Faradaic efficiency without being specifically optimized toward a single performance metric.

**FIGURE 4:**
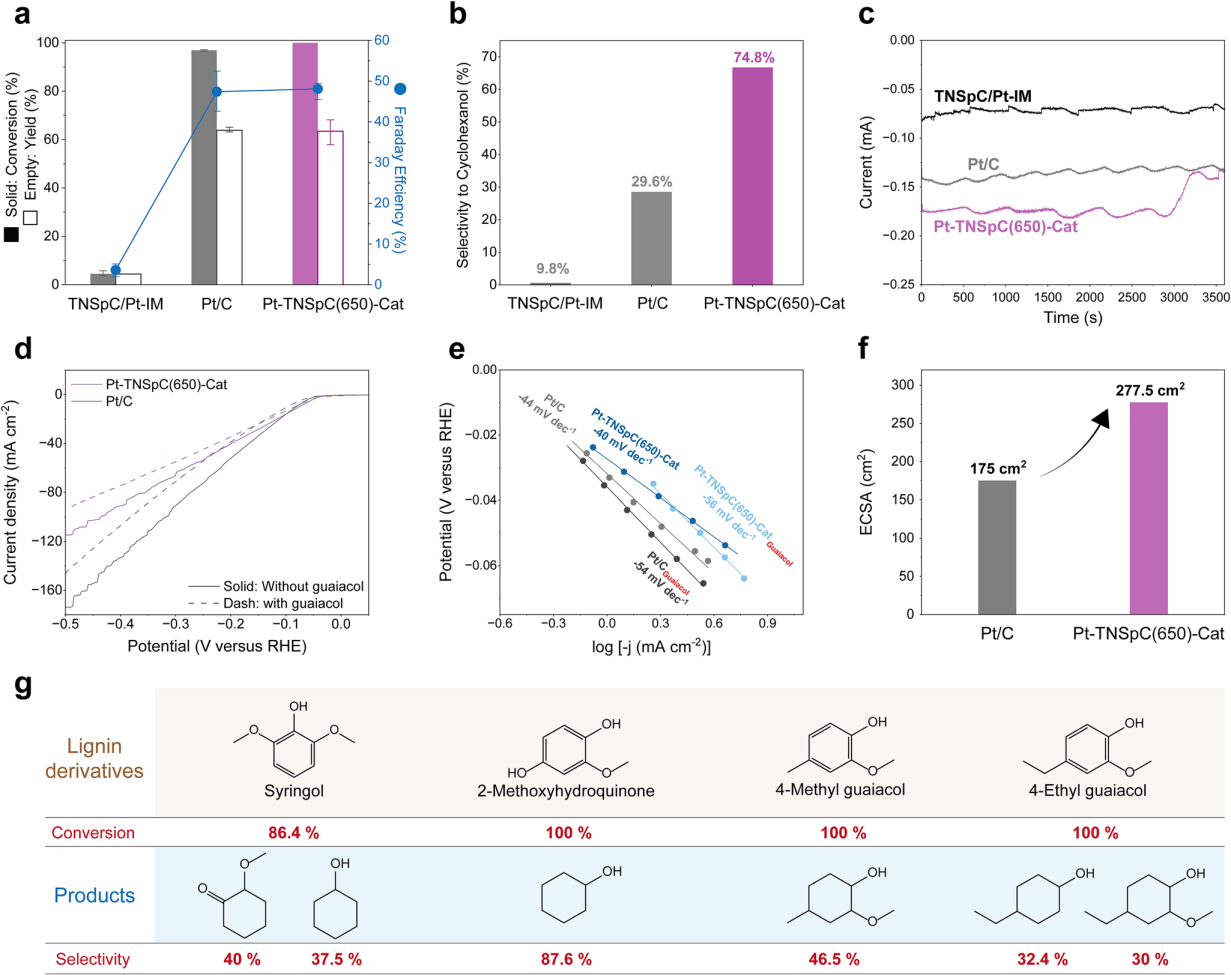
Electrocatalytic performance of Pt‐TNSpC. Performance of Pt‐TNSpC(650)‐Cat, commercial Pt/C and TNSpC/Pt‐IM for guaiacol ECH, including (a) Conversion, Yield and Faradaic efficiency, as well as (b) Selectivity to different reaction products. (c) Current density of different catalysts in guaiacol ECH. (d) Linear sweep voltammetry (LSV) curves with and without 25 mM guaiacol as the substrate, (e) Tafel slopes of Pt‐TNSpC(650)‐Cat and Pt/C calculated from the LSV curves. (f) Corresponding relative ECSA calculated from the double‐layer capacitance of catalysts at different potential scan rates (Figure S30). (g) ECH performance of different lignin derivatives, performed under identical reaction conditions. *Note*: The current densities reported in the LSV curves were normalized by the catalyst‐coated geometric area (6 cm^2^).

Tafel analysis further supports this interpretation. In the absence of guaiacol, Pt‐TNSpC(650)‐Cat and Pt/C show comparable Tafel slopes of 40 and 44 mV dec^−1^ (Figure 4e), suggesting that both follow a similar hydrogen evolution reaction (HER) mechanism. Upon introduction of guaiacol, variations in the Tafel slopes are observed for both catalysts (56 and 54 mV dec^−1^), consistent with a shift in the rate‐determining step associated with the transition from pure HER to guaiacol hydrogenation. These similarities indicate that the fundamental reaction pathway remains largely comparable, and that performance differences stem primarily from the greater accessibility and dispersion of active sites in Pt‐TNSpC(650)‐Cat, rather than from mechanistic differences. Moreover, we further demonstrated the general applicability of tannin catalyst across a range of lignin derivatives including syringol, 2‐methoxyhydroquinone, 4‐methyl guaiacol, and 4‐ethyl guaiacol. The obtained results demonstrated the general utility in the ECH reaction of lignin‐derived feedstocks (Figure 4g and Figure S31).

### 2.4 Benchmarked Environmental Impact of Pt‐TNSpC

Catalysts facilitate several chemical reactions, with minimized use of energy and resources, and are at the core of sustainable chemistry. However, their production tends to have a high environmental footprint. On that note, we quantified the environmental impacts associated to the production of our Pt‐TNSpC(650) catalyst via a prospective LCA assessment to obtain a cradle‐to‐gate climate change potential (Figures S34–S36, life cycle inventory provided in Tables S2–S4 for transparency). Proxy materials were used for tannins and PtCl_4_ as no secondary data could be found in the databases. Kraft lignin was used to replace tannin given their similarities in source materials and reported carbon footprints, while titanium tetrachloride was used to replace PtCl_4_ as both reagents have analogous synthetic routes with similar energy intensities [38, 39, 40]. Furthermore, the impacts from the most relevant metal catalyst for catalytic converters (palladium, platinum and rhodium) are provided for comparison.

Fractionation of 1 kg of condensed tannin and the complete development of 1 kg of tannin catalysts hold a carbon footprint of 233 and 1770 kg CO_2_‐eq.·kg^−1^, respectively, much below the 11510∼82660 kg CO_2_‐eq.·kg^−1^ footprint of conventional metal catalysts in the Ecoinvent v3.10 database, or the 13000∼80900 kg CO_2_‐eq.·kg^−1^ reported values in the current the literature (Figure 5a) [41]. While the probability distribution showed similar values (Figure S36), and overall standard deviations of *ca*. 5% and lower (Tables S5–S8), highlithing a robust assessement of the environmental impact of our tannin‐based catalyst. A plausible reason for the lower impact, despite the small scale of the process compared to that of commercial materials, may be the renewable origin and the reduced energy‐insensitivity of the production process, where the high‐temperature extraction and refinement processes from highly localized deposits are circumvented. Even if the inorganic catalysts are hypothetically retrieved from secondary resources (via integrated refining recovery plants), their carbon footprint remains certainly high (180 kg CO_2_‐eq.·kg^−1^ for Pt, 540 kg CO_2_‐eq.·kg^−1^ for Pd, 1550 kg CO_2_‐eq.·kg^−1^ for Rh).

**FIGURE 5:**
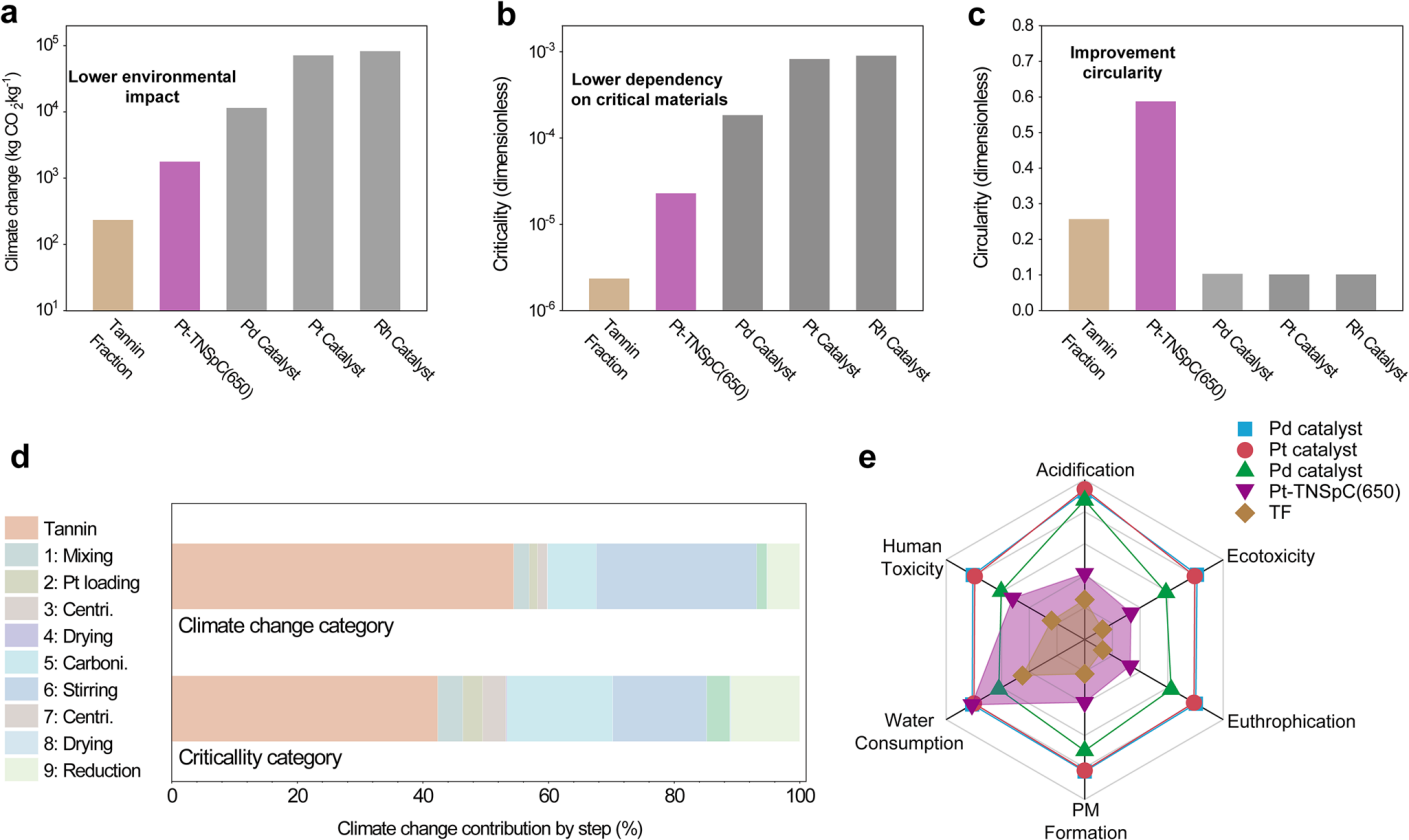
Environmental impact of the preparation of Pt‐TNSpC(650)‐Cat catalyst. Cradle‐to‐gate impacts for the categories of (a) climate change potential, (b) SH2E criticality, and (c) material circularity indicator (MCI). Climate change potential and criticality are represented using a logarithmic scale on the vertical axis. (d) Disaggregated cradle‐to‐gate climate change potential and criticality for the various steps utilized in the preparation of the tannin catalyst. (e) Radar plot of additional environmental impact categories comparing Pt‐tannin based catalyst and commercial Pt catalyst (radially increasing values from the center to the edge represent a greater environmental impact).

Material criticality, highly relevant in the current supply chain landscape, of our tannin‐based catalyst displayed value (2.29 × 10^−5^, dimesionless metric) up to two orders of magnitude lower compared to current precious metal catalysts (Pd, Pt, Rh) (Figure 5b). Furthermore, we assessed the circularity of the catalyst using the materials circularity indicator (MCI) to provide further insights on the supply chain and suitability of the tannin catalyst for its integration into the circular economy. By considering aspects related to materials flow (e.g., amount of virgin and recycled materials, generated waste), the MCI gives a value of 1 to a fully circular product system, while a fully linear product system is denoted with 0.1. Interestingly, the tannin catalyst shows a remarkable MCI of 0.587, while all the Pd, Pt, and Rh catalysis present nearly linear values, *ca*. 0.102 (Figure 5c).

We then disaggregated the contribution of individual steps taken during the tannin catalyst production on climate change and criticality categories (Figure 5d) [42]. Tannin fractionation is the most relevant driver in both cases (54.5% and 42.4% share, respectively) because of the need of high contents of acetonitrile. With a 25.5% contribution, stirring (step 6, high acetone use) is the second most impactful process in the category of climate change, while carbonization (16.8%, step 5) and stirring (15.0%, step 6) are the second and third most relevant drivers in terms of material criticality due to the large renewable energy consumption (requiring extensive infrastructure that relies on critical materials) [43] as well as acetone utilization. Importantly, the raw tannin acquisition (proxy material), as well as the Pt use for catalysts formation (step 2, proxy material), bear together 10.1% of the total climate change in the catalysts.

Our tannin‐based catalyst also showed additional benefits as far as other relevant impact categories (Figure 5e). Compared to the reference catalysts, our tannin‐based catalyst displayed notably better performance in acidification, freshwater ecotoxicity, freshwater eutrophication, particulate matter (PM) formation, water use, and carcinogenic human toxicity. For example, up to two orders of magnitude lower impacts are achieved in terms of acidification, eutrophication, or PM formation. However, it is worth noting the high values obtained in the water use category, where the tannin catalyst (18007 m^3^‐world eq.) equals the water needs of Pt and Rh catalysts. This enhanced water usage is a common trend observed in bio‐based product when compared to non‐renewable products [44]; therefore, this is a step that needs careful consideration in future tannin biorefineries.

## 3 Conclusions

Phenolic particles, and associated carbons, have been mostly made using phenolic resins. First efforts to make phenolic particles from natural precursors have used lignin; however, lignin is thermoplastic and not soluble in water. These two features limit particle making to those processes requiring organic solvents for lignin dissolution (e.g. THF), and extensive thermal stabilization periods when converting then into carbons. In our work, highly uniform phenolic particles are produced from condensed tannins in aqueous medium at room temperature, via a self‐assemble process that is initiated by DMAPA induced hydrogen bonding followed by hydrophobic interactions. The process is governed by low initial nucleus density from slow infusion, promoting ordered growth via surface energy minimization. We noted that tannin fractionation was essential for obtaining homogenized starting precursors for enabling uniform particles. Compared with conventional covalent polymerization routes, our method maximally preserves the abundant phenolic hydroxyl groups on the TNSp surface. These phenolic hydroxyl groups not only serve as electron donors for the subsequent in situ reduction of Pt^0^, but they also act as natural anchoring sites that enable uniform capture and controllable dispersion of metal ions through coordination or electrostatic interactions. This effectively suppresses metal migration and sintering during pyrolysis, yielding highly dispersed metal nanoparticles.

The surface of the nanospheres is highly populated by phenolic‐amine groups and allowed the precise structuring of Pt, Co, and Ni nanoparticles sizing 1–2 nm and with no sintering during pyrolysis. Surface‐structuring metal species on carbon particles is central for the preparation of catalysts. Here, the great dispersion and uniformity of the Pt species on the surface of the carbonized TNSp resulted in enhanced electrocatalytic hydrogenation of guaiacol when compared to commercial Pt/C, with high activity and selectivity. Of note, due to its high binding capacity, tannic acid is currently the flagship plant polyphenol for various metal‐binding purposes; however, they are a small fraction of the whole polyphenol biodiversity, and their feasible implementation depends greatly on the targeted field – tending to find more uses in the biomedical field due to its naturally higher added‐value [33].

Additionally, to its better electrocatalytic performance, our tannin‐based catalyst is also a more sustainable option compared to the platinum group metal catalysts in the area. Early‐stage LCA is not a final sustainability metric as the process is still open for further optimization in terms of chemical and energy use, but it provides a framework to evaluate the future potential impact of the catalyst while identifying current development areas to further improve its environmental performance. In our case, we identified the water usage as the critical parameter, and further studies should focus on the production of tannin particles in higher concentration with reduced water consumption. Nevertheless, optimizing the use of precious metals is key within the sustainable catalysis landscape given that precious metals are typically categorized under critical materials.

In summary, we demonstrate a circular design strategy that upcycles abundant condensed tannin (proanthocyanidins) into customizable functional nanospheres, exhibiting uniform structures and excellent performance in electrocatalytic applications. Given the large global use of synthetic phenolics in modern society, this work underscores the viability of plant‐based feedstocks for sustainable development. We expect this research will be a milestone in plant‐based polyphenols research, driving the sustainable utilization of biomass.

## 4 Experimental Section

### 4.1 Materials and Chemicals

Condensed tannin provided by TANAC S/A Brazil was used as a starting material. Acetonitrile (CH_3_CN, ≥ 99.0%, HPLC), 3‐(Dimethylamino)‐1‐propylamine ((CH_3_)_2_N(CH_2_)_3_NH_2_, 99%), and Platinum (IV) chloride (PtCl_4_, ≥ 96%) were purchased from Merck. Epigallocatechin Gallate Hydrate (EGCG, ≥ 98.0%), Ethylenediamine (EDA, anhydrous, ≥ 98.0%), Cobalt (II) chloride (CoCl_2_, anhydrous, ≥ 98.0%), Nickel (II) Chloride (NiCl_2_, anhydrous, ≥ 98.0%), and Acetone (C_3_H_6_O, ≥ 99.5%, GC) are purchased from TCI, Finland. Ethanol (CH_3_CH_2_OH, ≥ 99.5%) provided by Anora Group Oyj, Finland. Carbon felt (high‐temperature polyacrylonitrile (PAN)‐based carbon fiber felt provided by Beihai Carbon Co., Ltd.). All chemicals were used without further purification.

### 4.2 Fractionation of Condensed Tannin

Fractionation was performed using a 250 mL Soxhlet extraction system with acetonitrile as the solvent. 20 g of condensed tannin was placed in a cellulose extraction thimble (Whatman, Merck, Finland). Each fractionation process consisted of 8 cycles, where one cycle was defined as a complete siphoning event in the Soxhlet apparatus. The resulting product, designated as the tannin fraction (TF), was subsequently used for the tannin nanosphere synthesis.

### 4.3 Synthesis of Tannin Nanosphere (TNSp)

TNSp were synthesized using TF as the raw material and 3‐(dimethylamino)‐1‐propylamine (DMAPA) as a polymerization initiator. In a typical system, 840 mg of TF was first dissolved in 2 mL ethanol to form a orange color transparent solution (Figure S10a), which was then diluted with 78 mL deionized water (DW). Separately, 440 µL DMAPA was added to 80 mL DW to form DMAPA solution. Each solution was subsequently introduced via a peristaltic pump at a rate of 4 mL/min into a glass bottle containing 80 mL DW under moderate stirring (Figure S4). The resulting TNSp were washed thoroughly with water, collected by centrifugation, and dried under vacuum at 30°C for 12 h. The yield of TNSp was calculated by following equation:

(1) YieldTNSp(%)=MTNSp/MTF

where M_TF_ and M_TNSp_ are the weight of TF and TNSp (mg), respectively.

### 4.4 Synthesis of Pt‐Loaded TNSp (Pt‐TNSp)

The synthesis of Pt‐TNSp followed the same procedure as pristine TNSp, with an additional Pt loading step. Specifically, when the TNSp synthesis reaction reached 24 h, 10 mL solution containing 14 mg PtCl_4_ was added to the suspension and was rapid stirred for another 24 h. The product was then washed with water, collected by centrifugation, and dried under vacuum at 30°C for 12 h to obtain Pt‐TNSp.

### 4.5 Preparation of Tannin Catalyst (Pt‐TNSpC(650))

The obtained Pt‐TNSp were pyrolyzed under nitrogen atmosphere at 650°C with a heating rate of 5°C/min for 2 h, the yielding Pt‐TNSpC(650) was subsequently reduced under H_2_ atomsphere for further exploration.

### 4.6 Characterization

The morphology of each sample was observed with a field‐emission scanning electron microscope (SEM, Zeiss Sigma VP) and a field‐emission transmission electron microscope (FE‐TEM, JEOL JEM‐2200FS). ^1^H NMR and HSQC spectra were acquired using a Bruker NMR Spectrometer (AV NEO 400). XRD patterns were collected using a Rigaku SmartLab x‐ray diffractometer (40 kV, 30 mA, Cu Kα λ  =  1.5406 Å). SAXS measurements were taken using a Xenocs SAXS Xeuss 3.0 system with an x‐ray wavelength of λ  =  0.957 Å and a sample‐to‐detector distance of 55 mm. The absorption spectrum was measured by a UV–vis spectrometer from 200 to 500 nm (Shimadzu UV‐2550). The Pt concentration was collected on ICP‐OES. The molecular weight and distribution of condensed tannin were evaluated using a Gel Permeation Chromatography with an Agilent 1100 HPLC system equipped with a PSS MCX 300 × 8 mm column. The thermostability was measured by a thermogravimetric analyser (TGA 5500) using a heating rate of 5°C/min under N_2_. FTIR spectra were recorded using a Bruker FTIR ALPHA II spectrometer under the attenuated total reflectance (ATR) mode. XPS measurements were performed with a Kratos AXIS Ultra DLD x‐ray photoelectron spectrometer using a monochromated AlKα x‐ray source (1486.7 eV) run at 100 W with pass energy of 80 eV.

### 4.7 Electrocatalytic Analysis

Electrocatalytic hydrogenation of guaiacol was performed in a two‐compartment glass H‐cell separated by a DuPont Nafion‐117 membrane. Catholyte and Anolyte were both 25 mL and consisted of 0.5 m H_2_SO_4_ at pH = 0. A carbon felt (2 × 3 cm^2^) served as the working electrode, while a Pt foil and a Hg/Hg_2_SO_4_ electrode with a saturated aqueous K_2_SO_4_ solution filling were used as the counter and reference electrodes, respectively (Figure S32). The whole cell was placed in a water bath with controlled temperature. All electrochemical experiments were conducted with a Corrtest CS350M dual channel potentiostat, with the concentration of guaiacol fixed at 25 mm. All potentials (*E*) were converted to the RHE scale via the following equation:

(2) E(vs.RHE)=E(Hg/Hg2SO4)+0.658+0.0591×pH

### 4.8 Life Cycle Assessment (LCA)

The environmental impacts of tannin catalyst synthesis were quantified through a cradle‐to‐gate life cycle assessment (LCA), excluding equipment amortization. The life cycle inventory (LCI) was constructed according to primary laboratory data (Tables S1–S3 and Figure S33–S34) supplemented by the “ecoinvent v3.10 Cut‐Off” database, a mix of renewable medium‐voltage electricity was used as the energy source in the LCI. The calculations were performed utilizing the OpenLCA 2.4.0 software and 1 kg of material as the functional unit (fractionated tannin or catalyst).

## Conflicts of Interest

The authors declare no conflicts of interest.

## Supporting information




**Supporting File**: smll75461‐sup‐0001‐SuppMat.docx.

## Data Availability

The data that support the findings of this study are available from the corresponding author upon reasonable request.
