# Supplementary material for: Monodisperse Condensed Tannin and Derived Carbon Nanospheres Featuring Well‐Defined Surface‐Structured Metal Species
Source: Small. 2026 Aug 26;22(61):e75461. doi: 10.1002/smll.75461 (PMC13725408; doi:10.1002/smll.75461)
Supplement: Supplementary file 1 [file SMLL-22-e75461-s001.docx]

Supplementary Material

**Monodisperse Condensed Tannin and Derived Carbon Nanospheres Featuring Well-Defined Surface-Structured Metal Species**

*Weitong Wang,^1, #^ Jiaqi Wang,^2, #^ Bin Zhao,^1,3^ Tianyang Wen,^4^ Daniel Martín-Yerga,^2^ Monika Österberg,^1^ Erlantz Lizundia,^5^ and Bruno D. Mattos.^1, *^*

^*^Corresponding author: Bruno D. Mattos. e-mail: bruno.mattos@aalto.fi

^#^These authors contributed equally*.*

W. Wang, Dr. B. Zhao, Prof. M. Österberg, Prof. B. D. Mattos,

Department of Bioproducts and Biosystems, School of Chemical Engineering, Aalto University, Vuorimiehentie 1, Espoo FI-00076, Finland

E-mail: bruno.mattos@aalto.fi

J. Wang, Prof. D. Martín-Yerga

Department of Chemistry and Materials Science, School of Chemical Engineering, Aalto University, Espoo, FI-02150, Finland.

Dr. B. Zhao

Department of Applied Physics, School of Science, Aalto University, Espoo, FI-00076, Finland.

T. Wen

Department of Chemical and Metallurgical Engineering, School of Chemical Engineering, Aalto University, Espoo, FI-02150, Finland.

Prof. E. Lizundia

Life Cycle Thinking Group, Department of Graphic Design and Engineering Projects, Faculty of Engineering in Bilbao. University of the Basque Country (EHU), Bilbao 48013, Spain.

**This file contains 3 Sections, 35 Supplementary figures, 7 Supplementary tables in 50 pages**

**Section 1: Supplementary Methodology**

**Synthesis of TNSp**-**0.56x, TNSp**-**0.78x, and TNSp**-**1.22x**

In each procedure, 840 mg of TF was first dissolved in 2 mL ethanol to form a dark orange transparent solution, which was then diluted with 43, 60, and 96 mL DW, for TNSp-0.56x, TNSp-0.78x, and TNSp-1.22x, respectively. 440 µL DMAPA was also added to 45, 62, and 98 mL DW to form DMAPA solution, respectively. Each solution was subsequently introduced via a peristaltic pump at a rate of 4 mL/min into a glass bottle containing DW under moderate stirring, which finally form systems with volumes of 135, 188, and 293 mL. The resulting TNSp-0.56x, TNSp-0.78x, and TNSp-1.22x were washed thoroughly with water, collected by centrifugation, and dried under vacuum at 30 °C for 12 h.

**Synthesis of condensed tannin particles (CTPs), Tannin fraction particle (TF particle) and EGCG nanospheres (EGNSp)**

CTPs, TF particle, and EGNSp were synthesized following a same process. Firstly, condensed tannin, tannin fraction and EGCG were dissolved in EtOH to from a solution with concentration at 2 g/10 mL. Then, 250 µL tannin solutions was added in 7.75 mL DW followed with 13 µL DMAPA, then solution was mixed and stirring for 24 h. The resulting CTPs and TPs were washed thoroughly with water, collected by centrifugation, and dried under vacuum at 30 °C for 12 h.

**Synthesis of Co-TNSp and Ni-TNSp**

Co-TNSp and Ni-TNSp were prepared by replacing PtCl_4_ with 18 mg CoCl_2_ and 18 mg NiCl_2_, respectively, under otherwise identical conditions.

**Preparation of Pt-TNSpC(350), Pt-TNSpC(450), Pt-TNSpC(550), and Pt-TNSpC(750)**

The obtained Pt-TNSp were pyrolyzed under nitrogen atmosphere at 350, 450, 550, and 750 °C, respectively, with a heating rate of 5 °C/min and maintain for 2 h. Then, obtained Pt-TNSpC(350), Pt-TNSpC(450), Pt-TNSpC(550), and Pt-TNSpC(750). The yield of TNSpC(X) was calculated by following equation:

*Yield_TNSpC(X)_ (%) = M_TNSpC(X)_* / *M_TNSp_*	(1)

**Preparation of Co-TNSpC(650)and Ni-TNSpC(650)**

The obtained Co-TNSp and Ni-TNSp were pyrolyzed under nitrogen atmosphere at 650 °C, with a heating rate of 5 °C/min and maintain for 2 h. Then, obtained Co-TNSpC(650) and Ni-TNSpC(650).

**Preparation of catalyst TNSpC/Pt-IM**

The synthesis of TNSpC/Pt-IM was based on TNSp carbon (TNSpC). Firstly, TNSp was pyrolyzed under nitrogen atmosphere at 650 °C with a heating rate of 5 °C/min for 2 h, the yielding TNSpC(650) was then mixed with PtCl_4_ to make Pt concentration at around 5 wt% in a flask with water, following a rapid stirring combined with heat until evaporated to dryness, then reduced under H_2_ atmosphere for further exploration.

**Molecular dynamics simulation**

MD simulations were conducted using GROMACS software package (2020.6 version).^[1]^ The amber99sb-ildn force field were used to parametrize all atoms, such as the bond parameters, angle parameters and the dihedral angles, and so on. The RESP charges were calculated using ORCA^[2]^ at the B3LYP/6-311+g(d,p) level. TIP3P was used for the model of water molecule. The steepest descent method was applied to minimize the initial energy for each system with a force tolerance of 1 kJ/(mol^-1^ nm^-1^ ) and a maximum step size of 0.002 ps before MD calculations. In all the three directions, periodic boundary conditions were imposed. Leapfrog algorithm was used to integrate the Newtonian equation of motion. The MD simulation was processed in an NPT ensemble and the simulation time is 50 ns at 298.15 K (2.0 fs time step). In NPT simulations, the pressure was maintained at 1 bar by the Berendsen barostat in an isotropic manner. The Particle-Mesh-Ewald (PME) with a fourth-order interpolation was used to evaluate the electrostatic interactions and the grid spacing is 1.0 Å, whereas a cutoff of 1.0 Å was employed to calculate the short-range van der Waals interactions.

**Electrochemical analysis**

Linear sweep voltammetry (LSV) was performed in the aforementioned H-type cell over a potential window of 0 to -0.55 V (vs. RHE) at a scan rate of 20 mV s⁻¹. The electrochemical capacitance was evaluated by cyclic voltammetry (CV).

Initially, the non-Faradaic potential region was identified from the CV response, which generally corresponds to a 0.1 V window centered around the system’s open-circuit potential (OCP). Subsequently, CV experiments were carried out in a quiescent electrolyte by sweeping the potential within this non-Faradaic region from positive to negative limits and then reversing, at five different scan rates (0.5, 1, 2.5, 5, and 10 mV s⁻¹). At each turning point, the working electrode was maintained for 60 s before commencing the following sweep. The electrochemically active surface area (ECSA) was calculated using the relation:

ECSA = C_dl_/C_s_ (1)	(2)

Where the double-layer capacitance (C_dl_) was obtained by plotting Δj = (j_a_ − j_c_)/2, where j_a_ and j_c_ represent the anodic and cathodic current densities at a selected potential, as a function of the scan rate. The slope of the linear fit was taken as C_dl_. Cs denotes the specific capacitance of a smooth standard electrode with an actual surface area of 1 cm^2^, typically ranging from 20 to 60 μF cm^-2^. In this work, an average value of 40 μF cm^-2^ was adopted.

**Product analysis**

Concentration in the system was monitored by withdrawing 1 mL sample aliquots at prescribed time intervals from the cathode chamber. The samples were further saturated with NaCl and acidified to pH = 1 by HCl, then 3 mL ethyl acetate was added to extract the organics. The solution was filtrated using a 0.45 μm syringe filter for GC/MS analysis. All the samples were analyzed with a Shimadzu GC-MS-QP2010SE. The GC used an HP1 column, 30 m × 0.25 mm with a 0.25 μm film thickness, a 1.0 ml min-1 helium carrier gas flow rate, and a split ratio 1:5. The injector temperature was set at 260 °C. The GC oven program was started and stayed for 3 min at 45 °C, then the oven was heated at 15 °C min^-1^ to 250 °C. Mass spectrometry was operated in the electron ionization (EI) mode at an ionization energy of 80 eV, a m/z ranging from 28 to 400, and a sampling interval of 0.34 s. External standards were also used to identify compounds and quantify the peaks.

The conversion, selectivity, yield, and faradaic efficiency were calculated according to the following equations:

*Conversion (%) = (moles reactant consumed/initial moles reactant) × 100%	(3)*

*Selectivity (%) = (moles desired product/total moles products) × 100%	(4)*

*Yield (%) = (actual moles product/theoretical outputs) × 100%	(5)*

*Faradaic efficiency (%) = (Electrons used to generate products/Total electrons transferred)	(6)*

**Life cycle assessment**

The environmental impacts originating from the fractionation of the condensed tannins, and the subsequent catalysts preparation were determined in the light of the life cycle assessment (LCA) methodology. Given the unknown end-of-life treatment of the developed catalyst, as well as the current industry treatment for benchmark catalysts, this study adopts a *cradle-to-gate* system boundary,^[3]^ and covers the raw material acquisition with their upstream processes, the on-site energy consumption for lab-scale processing, and the management of the generated wastes during production (equipment amortization is left aside). The life cycle inventory (LCI) modeling, the second fundamental step during LCA (provided in **Tables S1~S3** and **Figures S33~S35**), was constructed according to primary laboratory data. For the secondary data required to complete the LCI, the *“ecoinvent v3.10 Cut-Off”* database was utilized. In light of the ongoing transition from fossil fuels to renewable energy through electrification,^[4]^ a mix of renewable medium-voltage electricity was used as the energy source in the LCI. The calculations were performed utilizing the OpenLCA 2.4.0 software and 1 kg of material as the functional unit (fractionated tannin or catalyst). As the third phase when conducting the LCA, the life cycle impact assessment (LCIA) was conducted in the light of the *Environmental Footprint v3.1* methodology, SH2E Criticality Indicator developed by Bargiacchi et al. and Zapp and Schreiber,^[5]^ and the Material Circularity Indicator (MCI) from the Ellen MacArthur Foundation and Ansys Granta.^[6]^ Briefly, the criticality is obtained as:

$Criticality= M_{n}\times{CF}_{m}$	(7)

where *CF* is a characterization factor based on the studied material *m*, and *M* is the amount of the given material. CF is obtained as:

$CF= \frac{SR}{C \times\left( 1-IR \times\left( 1-{EoL}_{RIR} \right) \right)}$	(8)

where *SR* is the supply risk (European Commission´s scaling), *C* is the material consumption, *IR* is the import reliance, and *EoL_RIR_* accounts for the recycling input rate. The MCI was determined based on the linear flow index (LFI) considering the flow of the materials, and the utility factor F(X) that takes into account the life time of the product compared to average benchmark:^[7]^

$MCI= 1-LFI \times F\left( x \right)$	(9)

where:

$LFI= \frac{V + W}{2M+ \frac{W_{F}- W_{C}}{2}}$ , $F\left( X \right)= \frac{0.9}{X}$, and	$X= \frac{L}{L_{av}} \times\frac{U}{U_{av}}$	(10)

where LFI is the linear flow index considering the amount of virgin material *V*, the waste *W*, and the mass *M* (_F_: to produce secondary material feedstock for secondary life material; _C_: form the recycling process). The combined analysis of the criticality and circularity, with special focus on the use phase (as opposed to other circularity metrics such as the Circularity Index by Cullen)^[8]^ offers a more comprehensive perspective in line with the current stringent environmental requirements, which focus not only on the carbon footprint but also on the bigger implications of material use.^[8a, 9]^ In addition, a Monte Carlo simulation with 2,000 iterations was done to assess potential uncertainties.

**Section 2: Supplementary Figures**

**Figure S1.** GPC curves of **a)** industrial condensed tannins (CT) and **b)** fractionated tannins (TF) reveal that fractionation reduces the molecular weight and increases the structural homogeneity, which have lower polydispersity index, PDI. **c)** Solubility of CT and TF in each solvent (water, MeOH, EtOH, Acetone), tannin powders were dissolved in each solvent for 24 hours, then centrifuge for 30 min at 11000 rpm.

**Figure S2.** Condensed tannin particles (CTPs) prepared from unfractionated tannins, which display a disordered surface morphology, resulting in poor monodispersity and uncontrolled particle sizes.

**Figure S3.** TF particle derived from fractionated condensed tannins show incipient monodispersity, yet possess a broad size distribution (from ~50 to ~800 nm) with poor uniformity.

**Figure S4.** Setup for TNSp preparation. A peristaltic pump simultaneously delivers TF and DMAPA solutions into a glass container under gentle stirring.

**Figure S5.** Tannin nanospheres (TNSp), which exhibits excellent uniformity and monodispersity following various adjustments.

**Figure S6.** The pH change monitored during TNSp preparation reflects the deprotonation process of the amine groups on DMAPA, which have pKa values of approximately 10.5 (primary) and 9.0 (tertiary).

**Figure S7. a)** Photographs showing the TNSp and **b)** TF particle assembly process at different times.

**Figure S8.** XPS spectra of **a)** TNSp with high resolution of **b)** carbon, **c)** oxygen, and **d)** nitrogen peaks.

**Figure S9.** FT-IR spectra of DMAPA, Tannin Fraction, and TNSp. The spectrum of TNSp appears largely unchanged compared to that of the Tannin Fraction, with the main difference being a peak broadening in the 3500~3000 cm⁻¹ region. The absence of a new amide (R−C(=O)−NR′R″, where R, R', and R″ represent any group, typically organic groups or hydrogen atoms.) peak in the 1700~1000 cm⁻¹ range indicates that DMAPA does not form covalent bonds with the tannins.

**Figure S10.** Thermogravimetric curves of TF and TNSp.

**Figure S11.** Photographs of **a)** tannin fractions (TF) and EGCG (from left to right) solutions (in EtOH), as well as **b)** TF solution mixed with DMAPA, EDA, and without amine (from left to right), and **c)** EGCG solution mixed with DMAPA, EDA, and without amine (from left to right) at 0 h, and **d), e)** after stirring for 24 h.

**Figure S12.** FT-IR spectra of DMAPA, epigallocatechin gallate (EGCG), and the nanospheres prepared from EGCG (EGNSp). The FT-IR spectrum of EGNSp exhibits a trend similar to that of the TNSp above.

**Figure S13.** SEM image of EGNSp, exhibits similar properties to TNSp.

**Figure S14.** ^1^H NMR spectra of **a)** TNSp and EGNSp, also with a comparison between TF and EGCG. The spectra indicate that TF and EGCG share highly similar structures. Additionally, the polymerization process is evidenced by the appearance of broad peaks in the nanosphere spectra, while the phenolic hydroxyl peak from 8~10 ppm range disappears due to deprotonation. The 2D HSQC spectra of **b)** TNSp and **d)** EGNSp show no cross-peaks in the broad signal regions, ruling out covalent bonding. Therefore, the broadness is attributed to the presence of free hydrogen protons or hydrogen bonds. **c)** Variable-temperature ¹H NMR spectra show the broad peak shifting high-field as temperature increased, this shift is attributed to the weakening of hydrogen bonds, thereby confirming the non-covalent character of the interaction.

**Figure S15. a)** DMAPA and **b)** EGCG molecular structures.

**Figure S16.** DMAPA and EGCG were simulated together in water at 298.15 K for **a)** 0 ns and **b)** 50 ns, the two components readily assembled into aggregates after 50 ns.

**Figure S17.** Schematic of the DMAPA/EGCG Aggregate at 50 ns (Visualized without Water Molecules)

**Figure S18. a)** Number of aggregated phenolic as a function of time. **b**) Number of hydrogen bonds in simulated EGCG-EDA systems, and **c)** evolution state of the model EGCG-EDA complex.

**Figure S19.** Uniform distribution of Pt species structured on the surface of Pt-TNSp.

**Figure S20.** EDS mapping spectra of Pt-TNSp.

**Figure S21.** TEM and EDS images of TNSp loaded with Co and Ni.

**Figure S22.** EDS mapping spectra of **a)** Co-TNSp and **b)** Ni-TNSp.

**Figure S23.** XPS spectra of **a)** Pt-TNSpC(650)-Cat as well as high resolution **b)** carbon, **c)** oxygen **d)** nitrogen, and **e)** platinum.

**Figure S24.** TEM images of Pt-TNSpC(X), obtained by pyrolyzing Pt-TNSp at different temperatures (X), show that the Pt nanoparticles remain uniformly dispersed without significant agglomeration.

**Figure S25.** **a**) Carbon yield after carbonization and Pt concentration. **b**) Evolution of particle size and size distribution in Pt-TNSpC(X) with different carbonization temperature.

**Figure S26.** XRD spectrum of Pt-TNSpC(X).

**Figure S27.** Evaluation of tannin catalysts (prepared at different pyrolysis temperatures) in guaiacol electrocatalytic hydrogenation, including **a)** conversion and yield, **b)** selectivity, **c)** current, and **d)** Faradaic efficiency (FE).

**Figure S28.** Evaluation of Pt-TNSpC(650)-Cat catalyst in guaiacol electrocatalytic hydrogenation, including **a)** conversion and yield, **b)** selectivity, **c)** current, and **d)** Faradaic efficiency (FE).

**Figure S29. a)** TEM and EDS images of TNSpC/Pt-IM catalyst prepared by impregnation method, shows the inhomogeneous dispersion of Pt species. And XPS spectra of b) high resolution platinum, **c)** low resolution TNSpC/Pt-IM as well as high resolution **d)** carbon, **e)** oxygen, and **f)** nitrogen.

**Figure S30.** Double-layer capacitances (Cdl) of the Pt-TNSpC(650)-Cat catalyst and reference Pt/C catalyst.

**Figure S31. a)** Conversion and **b**) Selectivity of the Pt-TNSpC(650)-Cat catalyst in electrocatalytic hydrogenation of different lignin model compounds.

**Figure S32.** Photographs of the H-type electrolysis cell used in the electrocatalysis experiments.

**Figure S33.** TEM image of Pt-TNSpC(650)-Cat after use.

**Figure S34.** Flowchart for the fractionation of condensed tannin. Material inputs are highlighted in pale green, electricity need is highlighted in yellow, processes are highlighted in blue, and wastes are highlighted in grey.

**Figure S35.** Flowchart for the fabrication of Pt-TNSpC(650)-Cat. Material inputs are highlighted in pale green, electricity need is highlighted in yellow, processes are highlighted in blue, gas need is highlighted in red, and wastes are highlighted in grey.

**Figure S36.** Monte Carlo LCA analysis for the category of *climate change*. For the sake of comparison, data corresponding to commercial metal catalyst for catalytic converters (palladium, platinum, and rhodium) are provided.

**Section 3: Supplementary Tables**

**Table S1.** Comparison of guaiacol electrocatalytic hydrogenation performance over the present catalyst, commercial Pt/C, and representative literature-reported catalysts.

|  | Reaction rate (mol·L^-1^·h^-1^·mg_metal_^-1^) | | Selectivity for Cyclohexanol (%) | Faradaic efficiency (%) | Temperature (^o^C) | Current density (mA·cm^-2^) |
| --- | --- | --- | --- | --- | --- | --- |
| Pt-TNSpC(650)-Cat (this work) | | 21.3 | 74.9 | 47 | 50 | 30 |
| Pt/C (this work) | | 20 | 29.6 | 46.8 | 50 | 25 |
| PtRhAu^[10]^ | | 0.48 | 11 | 69 | 20 | 200 |
| RhPtRu^[11]^ | | 0.8 | 8 | 70 | 25 | 50 |
| PtNiB/C^[12]^ | | 5 | 89 | 85 | 60 | 5 |
| Ru/CC^[13]^ | | 2 | 31 | 35 | 50 | 25 |
| PtCo_1_/MC^[14]^ | | 8 | 5 | 60 | 25 | 26 |
| PtRu/NC^[15]^ | | 3.5 | 78.5 | 65.4 | 80 | 200 |

**Note:** Because guaiacol hydrogenation involves multiple parallel and consecutive reaction pathways, different studies have generally pursued different optimization objectives. Some catalysts were designed to maximize Faradaic efficiency, whereas others were optimized toward a specific product. Consequently, conversion alone may not provide a sufficiently informative comparison, because it strongly depends on reaction time, initial substrate concentration, catalyst loading, and reactor configuration. We therefore used the overall reaction rate, normalized by the amount of metal catalyst, as a more quantitative activity metric than conversion alone.

Nevertheless, even the reported reaction rates cannot be regarded as strictly intrinsic or directly comparable because the applied potential/current density, temperature, electrolyte composition, and other operating conditions vary substantially among studies. In particular, temperature and the applied electrochemical driving force can markedly affect the observed reaction rate. We have therefore included the temperature and current density in the table to provide the necessary context and to avoid an oversimplified catalyst ranking.

As shown in **Table S1**, the reaction rate obtained in this work (21.3 mol·L^-1^·h^-1^·mgmetal^-1^) is comparable to, and slightly higher than, that of commercial Pt/C under similar conditions (20.0 mol·L^-1^·h^-1^·mgmetal^-1^). Meanwhile, our catalyst achieves a substantially higher cyclohexanol selectivity than commercial Pt/C (74.9% versus 29.6%), while maintaining a comparable Faradaic efficiency (47.0% versus 46.8%). Some literature catalysts exhibit higher Faradaic efficiencies or product selectivities, but generally at lower overall reaction rates or under substantially different temperatures and current densities.

We emphasize that our catalyst was not specifically optimized to maximize either cyclohexanol selectivity or Faradaic efficiency. Instead, its primary design objective was to achieve a high overall guaiacol hydrogenation rate comparable to that of commercial Pt/C. The simultaneously obtained cyclohexanol selectivity and Faradaic efficiency therefore demonstrate a favorable balance among activity, selectivity, and electron utilization.

**Table S2.** Material and energy inventory for “*Tannin fractionation”*. Wastes are highlighted in orange. Process output is highlighted in blue.

| **Comment** | **Item** | **Amount (g)** | **Source/provider** |
| --- | --- | --- | --- |
| ***Step 1: Fractionation*** | | | |
| *A proxy material is used* | Condensed tannin | 12.920 | *Kraft lignin from Ref:* [*https://advanced.onlinelibrary.wiley.com/doi/full/10.1002/adma.202416733*](https://advanced.onlinelibrary.wiley.com/doi/full/10.1002/adma.202416733) |
| *Solvent* | Acetonitrile | 31.44  (40 mL) | acrylonitrile production, Sohio process \| acetonitrile \| Cutoff, U - RER |
| *Electricity for heating* | Energy | 0.540 kWh | market for electricity, medium voltage, renewable energy products \| electricity, medium voltage, renewable energy products \| Cutoff, U - CH |
| ***Step 2: Evaporating*** | | | |
| Tannin fraction | | 0.840 | *From previous step* |
| *Electricity for evaporating* | Energy | 0.100 kWh | market for electricity, medium voltage, renewable energy products \| electricity, medium voltage, renewable energy products \| Cutoff, U - CH |
| *-* | Acetonitrile evaporation | 31.000 | Emission to air / unspecified |
| ***Step 3: Vacuum drying*** | | | |
| Tannin fraction | | 0.840 | *From previous step* |
| *Electricity for drying* | Energy | 0.020 kWh | market for electricity, medium voltage, renewable energy products \| electricity, medium voltage, renewable energy products \| Cutoff, U - CH |
| *-* | Acetonitrile evaporation | 0.440 | Emission to air / unspecified |
| **-** | **Tannin fraction** | **0.840** | ***Process output*** |

**Table S3.** Material and energy inventory for “Pt-TNSpC(650)-Cat”. Wastes are highlighted in orange. Process output is highlighted in blue.

| **Comment** | **Item** | **Amount (g)** | **Source/provider** |
| --- | --- | --- | --- |
| ***Step 1: mixing*** | | | |
| *Raw materials for nanoparticles* | Tannin fraction | 0.840 | *From previous step* |
| *-* | Dimethylaminopropylamine | 0.357 (440 µL) | dimethylaminopropylamine production \| dimethylaminopropylamine \| Cutoff, U - RER |
| *Solvent* | Water | 238.000 | water production, deionised \| water, deionised \| Cutoff, U - Europe without Switzerland |
|  | EtOH | 1.578 (2 mL) | ethanol production, ethylene hydration \| ethanol, without water, in 99.7% solution state, from ethylene \| Cutoff, U - RER |
| *Electricity for stirring* | Energy | 0.360 kWh | market for electricity, medium voltage, renewable energy products \| electricity, medium voltage, renewable energy products \| Cutoff, U - CH |
| *Electricity for* *pump* | Energy | 0.010 kWh | market for electricity, medium voltage, renewable energy products \| electricity, medium voltage, renewable energy products \| Cutoff, U - CH |
| ***Step 2: Pt loading*** | | | |
| *Raw materials for Pt loading* | Tannin nanospheres (TNSp) | 0.390 | *From previous step* |
| *A proxy material is used* | Platinum (IV) chloride | 0.014 | titanium tetrachloride production \| titanium tetrachloride \| Cutoff, U - RoW |
| *Electricity for stirring* | Energy | 0.360 kWh | market for electricity, medium voltage, renewable energy products \| electricity, medium voltage, renewable energy products \| Cutoff, U - CH |
| ***Step 3: centrifugation*** | | | |
| Pt loaded TNSp (Pt-TNSp) | | 0.390 | *From previous step* |
| *Electricity for centrifugation* | Energy | 0.430 kWh | market for electricity, medium voltage, renewable energy products \| electricity, medium voltage, renewable energy products \| Cutoff, U - CH |
| *Water + EtOH waste* | Wastewater | 239.000 mL | Wastewater, average |
| ***Step 4: Vacuum drying*** | | | |
| Pt-TNSp | | 0.390 | *From previous step* |
| *Electricity for drying* | Energy | 0.020 kWh | market for electricity, medium voltage, renewable energy products \| electricity, medium voltage, renewable energy products \| Cutoff, U - CH |
|  | Remaining water evaporation | 0.789 | Emission to air / unspecified |
| ***Step 5: carbonization*** | | | |
| Pt-TNSp | | 0.390 | *From previous step* |
| *Energy for heating* | Energy | 1.950 kWh | market for electricity, medium voltage, renewable energy products \| electricity, medium voltage, renewable energy products \| Cutoff, U - CH |
| *Inert gas* | N_2_ | 15.390 (12.300 L) | venting of nitrogen, liquid \| venting of nitrogen, liquid \| Cutoff, U - RER |
| ***Step 6: stirring*** | | | |
| Pt-TNSpC(650) | | 0.203 | *From previous step* |
| *Solvent* | Acetone | 31.360 (40 mL) | acetone production, from isopropanol \| acetone, liquid \| Cutoff, U - RER |
| *Electricity for stirring* | Energy | 0.090 kWh | market for electricity, medium voltage, renewable energy products \| electricity, medium voltage, renewable energy products \| Cutoff, U - CH |
| ***Step 7: centrifugation*** | | | |
| Pt-TNSpC(650) | | 0.203 | *From previous step* |
| *Electricity for centrifugation* | Energy | 0.430 kWh | market for electricity, medium voltage, renewable energy products \| electricity, medium voltage, renewable energy products \| Cutoff, U - CH |
| *Incineration as the most likely EoL treatment* | Acetone | 31.000 | process-specific burdens, hazardous waste incineration plant \| process-specific burdens, hazardous waste incineration plant \| Cutoff, U - RoW |
| ***Step 8: Vacuum drying*** | | | |
| Pt-TNSpC(650) | | 0.203 | *From previous step* |
| *Electricity for drying* | Energy | 0.020 kWh | market for electricity, medium voltage, renewable energy products \| electricity, medium voltage, renewable energy products \| Cutoff, U - CH |
| *-* | Remaining acetone evaporation | 0.360 | Emission to air / unspecified |
| ***Step 9: Reduce in H_2_/N_2_*** | | | |
| Pt-TNSpC(650)-Cat | | 0.203 | *From previous step* |
| *Energy for heating* | Energy | 1.260 kWh | market for electricity, medium voltage, renewable energy products \| electricity, medium voltage, renewable energy products \| Cutoff, U - CH |
| *Gas* | N_2_ | 11.485 (9.180 L) | venting of nitrogen, liquid \| venting of nitrogen, liquid \| Cutoff, U - RER |
|  | H_2_ | 0.092 (1.020 L) | hydrogen production, gaseous, petroleum refinery operation \| hydrogen, gaseous, low pressure \| Cutoff, U - Europe without Switzerland |
|  | H_2_ | 0.092 (1.020 L) | Emission to air / unspecified |
|  | **Catalyst (**Pt-TNSpC(650)-Cat**)** | **0.203** | ***Process output*** |

**Table S4.** Benchmark catalyst list.

| **Item** | **Amount (kg)** | **Source/provider** |
| --- | --- | --- |
| Rhodium | 1.000 | rhodium to generic market for metal catalyst for catalytic converter \| metal catalyst for catalytic converter \| Cutoff, U - GLO |
| Platinum | 1.000 | platinum to generic market for metal catalyst for catalytic converter \| metal catalyst for catalytic converter \| Cutoff, U - GLO |
| Palladium | 1.000 | palladium to generic market for metal catalyst for catalytic converter \| metal catalyst for catalytic converter \| Cutoff, U - GLO |

**Table S5.** Uncertainty analysis through the Monte Carlo method using the Environmental Footprint v3.1 methodology for 1 kg of Tannin catalysts. ^*^SD = standard deviation.

| **Impact category** | **Reference unit** | **Mean** | **SD^*^** | **5% Percentile** | **95% Percentile** |
| --- | --- | --- | --- | --- | --- |
| *Climate change: biogenic* | kg CO_2_-Eq | 81.44446814 | 24.47218496 | 50.46652835 | 125.4853 |
| *Climate change: fossil* | kg CO_2_-Eq | 1752.84931 | 89.82780895 | 1616.377172 | 1909.439 |
| *Human toxicity: carcinogenic* | CTUh | 1.19339E-05 | 2.32121E-06 | 8.57735E-06 | 1.6E-05 |
| *Human toxicity: carcinogenic, organics* | CTUh | 1.15601E-05 | 2.30167E-06 | 8.2433E-06 | 1.56E-05 |
| *Water use* | m^3^ world Eq deprived | 18056.80033 | 3293.879319 | 13193.65736 | 24087.14 |
| *Ecotoxicity: freshwater, inorganics* | CTUe | 12858.54127 | 1175.882498 | 11153.22083 | 14910.78 |
| *Eutrophication: freshwater* | kg P-Eq | 0.514350138 | 0.205018456 | 0.305854022 | 0.85417 |
| *Human toxicity: non-carcinogenic, inorganics* | CTUh | 2.43984E-05 | 7.43778E-06 | 1.5525E-05 | 3.79E-05 |
| *Human toxicity: carcinogenic, inorganics* | CTUh | 3.73894E-07 | 8.18659E-08 | 2.65343E-07 | 5.19E-07 |
| ***Climate change*** | **kg CO_2_-Eq** | **1836.211844** | **93.83973806** | **1696.614527** | **2000.228** |
| *Photochemical oxidant formation: human health* | kg NMVOC-Eq | 10.1814899 | 0.856411369 | 8.90385025 | 11.69698 |
| *Eutrophication: terrestrial* | mol N-Eq | 23.47568998 | 2.189674013 | 20.30218108 | 27.52074 |
| *Land use* | dimensionless | 233681.9377 | 99115.63568 | 110247.953 | 406521.5 |
| *Climate change: land use and land use change* | kg CO_2_-Eq | 1.918066405 | 0.350732453 | 1.467091014 | 2.548609 |
| *Eutrophication: marine* | kg N-Eq | 4.603079067 | 0.532650767 | 3.878571982 | 5.640541 |
| *Acidification* | mol H^+^-Eq | 11.72552729 | 0.743137923 | 10.63228886 | 12.9951 |
| *Ecotoxicity: freshwater* | CTUe | 19699.03538 | 2495.286933 | 16370.94772 | 24099.39 |
| *Ionising radiation: human health* | kBq U235-Eq | 133.3667095 | 129.2219172 | 48.06415206 | 359.401 |
| *Human toxicity: non-carcinogenic* | CTUh | 2.74589E-05 | 7.61106E-06 | 1.82995E-05 | 4.13E-05 |
| *Ecotoxicity: freshwater, organics* | CTUe | 6840.494114 | 1933.226197 | 4497.956501 | 10297.11 |
| *Human toxicity: non-carcinogenic, organics* | CTUh | 3.06048E-06 | 2.99821E-07 | 2.64577E-06 | 3.62E-06 |
| *Ozone depletion* | kg CFC-11-Eq | 6.12966E-05 | 6.36502E-06 | 5.2275E-05 | 7.26E-05 |
| *Energy resources: non-renewable* | MJ, net calorific value | 34449.10016 | 1739.453451 | 31719.85936 | 37464.39 |
| *Particulate matter formation* | disease incidence | 9.18741E-05 | 9.59175E-06 | 7.91392E-05 | 0.00011 |
| *Material resources: metals/minerals* | kg Sb-Eq | 0.027065881 | 0.006380931 | 0.018599931 | 0.038329 |

**Table S6.** Uncertainty analysis through the Monte Carlo method using the Environmental Footprint v3.1 methodology for 1 kg of Pt catalysts. ^*^SD = standard deviation.

| **Impact category** | **Reference unit** | **Mean** | **SD^*^** | **5% Percentile** | **95% Percentile** |
| --- | --- | --- | --- | --- | --- |
| *Climate change: biogenic* | kg CO_2_-Eq | 36.94498151 | 6.411235024 | 28.34235957 | 48.31957 |
| *Climate change: fossil* | kg CO_2_-Eq | 71162.94073 | 2741.739234 | 67226.90821 | 76222.21 |
| *Human toxicity: carcinogenic* | CTUh | 0.00014839 | 3.1484E-05 | 0.000105183 | 0.000203 |
| *Human toxicity: carcinogenic, organics* | CTUh | 0.000137388 | 3.08759E-05 | 9.52654E-05 | 0.000191 |
| *Water use* | m^3^ world Eq deprived | 15706.45419 | 1363.562526 | 13713.97558 | 18124.95 |
| *Ecotoxicity: freshwater, inorganics* | CTUe | 1453155.492 | 359921.8918 | 977880.401 | 2128957 |
| *Eutrophication: freshwater* | kg P-Eq | 88.78227493 | 40.12802305 | 44.39559521 | 157.2128 |
| *Human toxicity: non-carcinogenic, inorganics* | CTUh | 0.000888962 | 0.000596274 | 0.000412416 | 0.001883 |
| *Human toxicity: carcinogenic, inorganics* | CTUh | 1.10023E-05 | 5.15047E-06 | 6.64222E-06 | 1.97E-05 |
| ***Climate change*** | **kg CO_2_-Eq** | **71277.79582** | **2748.836388** | **67326.19281** | **76360.09** |
| *Photochemical oxidant formation: human health* | kg NMVOC-Eq | 1438.028655 | 126.8324832 | 1246.315136 | 1659.71 |
| *Eutrophication: terrestrial* | mol N-Eq | 5792.934003 | 626.751112 | 4861.699006 | 6870.457 |
| *Land use* | dimensionless | 605721.0628 | 27350.67115 | 566560.4436 | 654322.5 |
| *Climate change: land use and land use change* | kg CO_2_-Eq | 77.91010761 | 5.858615973 | 68.87420839 | 88.12736 |
| *Eutrophication: marine* | kg N-Eq | 395.694949 | 45.67466569 | 328.3296576 | 475.026 |
| *Acidification* | mol H^+^-Eq | 5024.014684 | 436.5821615 | 4380.566679 | 5804.96 |
| *Ecotoxicity: freshwater* | CTUe | 1497753.293 | 360314.3656 | 1020433.055 | 2171058 |
| *Ionising radiation: human health* | kBq U235-Eq | 2579.52351 | 2930.927412 | 993.2052993 | 6429.871 |
| *Human toxicity: non-carcinogenic* | CTUh | 0.000966556 | 0.000596305 | 0.000488327 | 0.001956 |
| *Ecotoxicity: freshwater, organics* | CTUe | 44597.80003 | 8062.596853 | 32856.01909 | 58940.14 |
| *Human toxicity: non-carcinogenic, organics* | CTUh | 7.7594E-05 | 1.15701E-05 | 6.19639E-05 | 9.83E-05 |
| *Ozone depletion* | kg CFC-11-Eq | 0.000458549 | 3.17783E-05 | 0.000409658 | 0.000513 |
| *Energy resources: non-renewable* | MJ, net calorific value | 1051165.983 | 98448.32777 | 901411.8218 | 1218803 |
| *Particulate matter formation* | disease incidence | 0.012381985 | 0.001696528 | 0.010147801 | 0.015526 |
| *Material resources: metals/minerals* | kg Sb-Eq | 3.527952582 | 0.050126654 | 3.467017971 | 3.62545 |

**Table S7.** Uncertainty analysis through the Monte Carlo method using the Environmental Footprint v3.1 methodology for 1 kg of Pd catalysts. ^*^SD = standard deviation.

| **Impact category** | **Reference unit** | **Mean** | **SD^*^** | **5% Percentile** | **95% Percentile** |
| --- | --- | --- | --- | --- | --- |
| *Climate change: biogenic* | kg CO_2_-Eq | 6.996629787 | 1.158666512 | 5.365488003 | 9.086527 |
| *Climate change: fossil* | kg CO_2_-Eq | 11496.03086 | 431.328887 | 10875.13709 | 12242.53 |
| *Human toxicity: carcinogenic* | CTUh | 2.57266E-05 | 5.26722E-06 | 1.83989E-05 | 3.55E-05 |
| *Human toxicity: carcinogenic, organics* | CTUh | 2.36897E-05 | 5.18429E-06 | 1.65571E-05 | 3.34E-05 |
| *Water use* | m^3^ world Eq deprived | 3016.670126 | 244.5069941 | 2637.827041 | 3434.942 |
| *Ecotoxicity: freshwater, inorganics* | CTUe | 215124.3227 | 52837.78953 | 146486.8947 | 312309.9 |
| *Eutrophication: freshwater* | kg P-Eq | 13.25230538 | 5.837397864 | 6.906614517 | 23.17233 |
| *Human toxicity: non-carcinogenic, inorganics* | CTUh | 0.000156605 | 8.04716E-05 | 8.45113E-05 | 0.000298 |
| *Human toxicity: carcinogenic, inorganics* | CTUh | 2.03696E-06 | 7.0588E-07 | 1.3656E-06 | 3.26E-06 |
| ***Climate change*** | **kg CO_2_-Eq** | **11516.97494** | **432.553984** | **10894.83629** | **12265.99** |
| *Photochemical oxidant formation: human health* | kg NMVOC-Eq | 304.8756653 | 24.55334202 | 267.3201696 | 347.0287 |
| *Eutrophication: terrestrial* | mol N-Eq | 832.428175 | 88.31309992 | 702.3461793 | 993.624 |
| *Land use* | dimensionless | 90971.34988 | 4506.858491 | 84127.24949 | 98823.9 |
| *Climate change: land use and land use change* | kg CO_2_-Eq | 13.94745027 | 0.993381709 | 12.39676341 | 15.58179 |
| *Eutrophication: marine* | kg N-Eq | 57.24357565 | 6.573179307 | 47.67058911 | 68.95681 |
| *Acidification* | mol H^+^-Eq | 2283.108087 | 283.1212155 | 1858.253456 | 2780.619 |
| *Ecotoxicity: freshwater* | CTUe | 222632.4944 | 52877.85711 | 154481.1252 | 320089.4 |
| *Ionising radiation: human health* | kBq U235-Eq | 624.8550099 | 574.211129 | 251.9756613 | 1520.564 |
| *Human toxicity: non-carcinogenic* | CTUh | 0.000182343 | 8.08626E-05 | 0.000107719 | 0.000323 |
| *Ecotoxicity: freshwater, organics* | CTUe | 7508.171773 | 1359.876412 | 5616.028255 | 10023.73 |
| *Human toxicity: non-carcinogenic, organics* | CTUh | 2.5738E-05 | 7.50712E-06 | 1.62258E-05 | 3.9E-05 |
| *Ozone depletion* | kg CFC-11-Eq | 8.37861E-05 | 5.91331E-06 | 7.48851E-05 | 9.4E-05 |
| *Energy resources: non-renewable* | MJ, net calorific value | 169571.7092 | 13889.02227 | 148806.0203 | 193092.7 |
| *Particulate matter formation* | disease incidence | 0.002977184 | 0.000314085 | 0.002509423 | 0.003526 |
| *Material resources: metals/minerals* | kg Sb-Eq | 0.759066067 | 0.009804333 | 0.746610755 | 0.776984 |

**Table S8.** Uncertainty analysis through the Monte Carlo method using the Environmental Footprint v3.1 methodology for 1 kg of Rh catalysts. ^*^SD = standard deviation.

| **Impact category** | **Reference unit** | **Mean** | **SD^*^** | **5% Percentile** | **95% Percentile** |
| --- | --- | --- | --- | --- | --- |
| *Climate change: biogenic* | kg CO_2_-Eq | 41.86437973 | 7.419469156 | 31.64599596 | 55.50173 |
| *Climate change: fossil* | kg CO_2_-Eq | 82494.3117 | 3222.41689 | 78045.29522 | 88444.84 |
| *Human toxicity: carcinogenic* | CTUh | 0.00017031 | 3.73474E-05 | 0.000118168 | 0.000239 |
| *Human toxicity: carcinogenic, organics* | CTUh | 0.000157855 | 3.67048E-05 | 0.000106897 | 0.000227 |
| *Water use* | m^3^ world Eq deprived | 17731.18393 | 1591.111224 | 15388.48052 | 20574.94 |
| *Ecotoxicity: freshwater, inorganics* | CTUe | 1706925.377 | 417269.0724 | 1159933.346 | 2501950 |
| *Eutrophication: freshwater* | kg P-Eq | 103.2828992 | 44.75143809 | 51.96031421 | 182.9081 |
| *Human toxicity: non-carcinogenic, inorganics* | CTUh | 0.001010016 | 0.00064767 | 0.000467642 | 0.00211 |
| *Human toxicity: carcinogenic, inorganics* | CTUh | 1.24551E-05 | 5.64222E-06 | 7.35931E-06 | 2.21E-05 |
| ***Climate change*** | **kg CO_2_-Eq** | **82625.05755** | **3231.14989** | **78171.13233** | **88601.04** |
| *Photochemical oxidant formation: human health* | kg NMVOC-Eq | 1594.502187 | 148.9210227 | 1373.461096 | 1850.217 |
| *Eutrophication: terrestrial* | mol N-Eq | 6866.006966 | 736.5806609 | 5758.516546 | 8149.048 |
| *Land use* | dimensionless | 710249.893 | 33250.07839 | 663425.411 | 767920.5 |
| *Climate change: land use and land use change* | kg CO_2_-Eq | 88.88146778 | 7.214034497 | 78.02825258 | 100.8928 |
| *Eutrophication: marine* | kg N-Eq | 469.3619293 | 54.06236701 | 388.8239613 | 565.4383 |
| *Acidification* | mol H^+^-Eq | 4239.100597 | 284.8146362 | 3795.211361 | 4714.168 |
| *Ecotoxicity: freshwater* | CTUe | 1758595.213 | 417489.26 | 1211244.317 | 2552786 |
| *Ionising radiation: human health* | kBq U235-Eq | 2751.048554 | 2858.521268 | 1007.576503 | 7105.243 |
| *Human toxicity: non-carcinogenic* | CTUh | 0.001085478 | 0.000647878 | 0.00053903 | 0.002186 |
| *Ecotoxicity: freshwater, organics* | CTUe | 51669.83571 | 9775.150653 | 37723.746 | 69385.45 |
| *Human toxicity: non-carcinogenic, organics* | CTUh | 7.54624E-05 | 8.13997E-06 | 6.41985E-05 | 8.97E-05 |
| *Ozone depletion* | kg CFC-11-Eq | 0.000519275 | 3.60962E-05 | 0.000464953 | 0.000583 |
| *Energy resources: non-renewable* | MJ, net calorific value | 1216245.559 | 114130.2107 | 1038406.622 | 1419325 |
| *Particulate matter formation* | disease incidence | 0.013247995 | 0.001962672 | 0.010659286 | 0.01675 |
| *Material resources: metals/minerals* | kg Sb-Eq | 3.882567796 | 0.05621259 | 3.81107013 | 3.987339 |

**References**

[1]	aH. J. C. Berendsen, D. v. d. Spoel, R. v. Drunen, *Computer Physics Communications* **1995**, *91*, 43–56; bM. J. Abraham, T. Murtola, R. Schulz, S. Páll, J. C. Smith, B. Hess, E. Lindahl, *SoftwareX* **2015**, *1-2*, 19–25.

[2]	F. Neese, *WIREs Computational Molecular Science* **2011**, *2*, 73–78.

[3]	aM. Iturrondobeitia, C. Vallejo, M. Berroci, O. Akizu-Gardoki, R. Minguez, E. Lizundia, *ACS Sustainable Chemistry & Engineering* **2022**, *10*, 9798–9810; bK. Bisaria, C. S. Seth, R. Singh, *Environmental Science: Advances* **2024**, *3*, 1153–1162.

[4]	Y. Yang, S. Xia, P. Huang, J. Qian, *Energy Strategy Reviews* **2024**, *52*.

[5]	openLCA, in *Databases, Life Cycle Impact Assessment* (Ed.: J. Cilleruelo), <https://www.openlca.org/new-calculate-material-criticality-with-openlca/>, **2024**.

[6]	J. Cilleruelo Palomero, L. Freboeuf, A. Ciroth, G. Sonnemann, *Cleaner Environmental Systems* **2024**, *12*.

[7]	E. M. Foundation, **2019**.

[8]	aB. Corona, L. Shen, D. Reike, J. Rosales Carreón, E. Worrell, *Resources, Conservation and Recycling* **2019**, *151*; bJ. M. Cullen, *Journal of Industrial Ecology* **2017**, *21*, 483–486.

[9]	aE. Commission, <https://single-market-economy.ec.europa.eu/sectors/raw-materials/areas-specific-interest/critical-raw-materials_en#fifth-list-2023-of-critical-raw-materials-for-the-eu>, **2024**; bJ. Huang, S. Wang, J. Chen, C. Chen, E. Lizundia, *Adv. Mater.* **2025**, *37*, e2416733.

[10]	T. Peng, T. Zhuang, Y. Yan, J. Qian, G. R. Dick, J. Behaghel de Bueren, S.-F. Hung, Y. Zhang, Z. Wang, J. Wicks, *J. Am. Chem. Soc.* **2021**, *143*, 17226–17235.

[11]	M. Wang, T. Peng, C. Yang, B. Liang, H. Chen, M. Kumar, Y. Zhang, W. Zhao, *Green Chem.* **2022**, *24*, 142–146.

[12]	Y. Zhou, Y. Gao, X. Zhong, W. Jiang, Y. Liang, P. Niu, M. Li, G. Zhuang, X. Li, J. Wang, *Adv. Funct. Mater.* **2019**, *29*, 1807651.

[13]	Z. Li, M. Garedew, C. H. Lam, J. E. Jackson, D. J. Miller, C. M. Saffron, *Green Chem.* **2012**, *14*, 2540–2549.

[14]	R. Wu, Q. Meng, J. Yan, Z. Zhang, B. Chen, H. Liu, J. Tai, G. Zhang, L. Zheng, J. Zhang, *Nat. Catal.* **2024**, *7*, 702–718.

[15]	Q. Zhou, R. Chen, Y. Chen, Y. Sun, J. Yuan, J. Xu, X. Liao, B. Shi, X. Xiao, *Industrial Crops and Products* **2025**, *229*, 120977.
